# MRL/MpJ Mice Resist to Age-Related and Long-Term Ovariectomy-Induced Bone Loss: Implications for Bone Regeneration and Repair

**DOI:** 10.3390/ijms24032396

**Published:** 2023-01-25

**Authors:** Xueqin Gao, Xuying Sun, Haizi Cheng, Joseph J. Ruzbarsky, Michael Mullen, Matthieu Huard, Johnny Huard

**Affiliations:** 1 Linda and Mitch Hart Center for Regenerative and Personalized Medicine, Steadman Philippon Research Institute, Vail, CO 81657, USA; 2 Department of Orthopaedic Surgery, McGovern Medical School, University of Texas Health Science Center at Houston, Houston, TX 77054, USA; 3 The Steadman Clinic, Vail, CO 81657, USA

**Keywords:** MRL/MpJ mice, aging, bone loss, ovariectomy, serum markers, osteoporosis

## Abstract

Osteoporosis and age-related bone loss increase bone fracture risk and impair bone healing. The need for identifying new factors to prevent or treat bone loss is critical. Previously, we reported that young MRL/MpJ mice have superior bone microarchitecture and biomechanical properties as compared to wild-type (WT) mice. In this study, MRL/MpJ mice were tested for resistance to age-related and long-term ovariectomy-induced bone loss to uncover potential beneficial factors for bone regeneration and repair. Bone tissues collected from 14-month-old MRL/MpJ and C57BL/6J (WT) mice were analyzed using micro-CT, histology, and immunohistochemistry, and serum protein markers were characterized using ELISAs or multiplex assays. Furthermore, 4-month-old MRL/MpJ and WT mice were subjected to ovariectomy (OV) or sham surgery and bone loss was monitored continuously using micro-CT at 1, 2, 4, and 6 months (M) after surgery with histology and immunohistochemistry performed at 6 M post-surgery. Sera were collected for biomarker detection using ELISA and multiplex assays at 6 M after surgery. Our results indicated that MRL/MpJ mice maintained better bone microarchitecture and higher bone mass than WT mice during aging and long-term ovariectomy. This resistance of bone loss observed in MRL/MpJ mice correlated with the maintenance of higher OSX^+^ osteoprogenitor cell pools, higher activation of the pSMAD5 signaling pathway, more PCNA^+^ cells, and a lower number of osteoclasts. Systemically, lower serum RANKL and DKK1 with higher serum IGF1 and OPG in MRL/MpJ mice relative to WT mice may also contribute to the maintenance of higher bone microarchitecture during aging and less severe bone loss after long-term ovariectomy. These findings may be used to develop therapeutic approaches to maintain bone mass and improve bone regeneration and repair due to injury, disease, and aging.

## 1. Introduction

Fracture non-unions and segmental bone defects remain challenging clinical dilemmas [1,2]. Furthermore, osteoporosis and its associated fracture risk significantly affect the life quality of the aged population [3,4]. Therefore, identifying new anabolic factors for bone growth or repair that can be used for the development of new therapies to treat osteoporosis and enhance bone repair is critical. 

Murphy Roths Large/lymphoproliferation (lpr) strain (MRL/MpJ) mice, also known as “super healer mice”, have demonstrated enhanced repair capacities for an array of musculoskeletal tissue injuries when compared to normal mice, including ear and skin wounds, nerve injuries, and articular cartilage/tendinous lesions [5,6,7,8,9,10,11,12,13,14,15]. Furthermore, MRL/MpJ mice have been shown to be relatively protected from post-traumatic arthritis after intra-articular fracture [16]. The underlying molecular mechanisms of enhanced tissue regeneration are attributed to modified inflammatory reactions, decreased fibrosis while improving remodeling [5,7,15,17], reduced cell apoptosis, increased cell proliferation/differentiation [7,18], and enhanced stem cell function [18,19]. In addition, the up-regulation of genes involved in repair processes, such as angiogenesis, deoxyribonucleic acid (DNA) repair and replication, protein synthesis, glycolysis, and cell adhesion have been reported in MRL/MpJ mice [7,15]. A recent investigation demonstrated an association between enhanced ear wound healing in MRL/MpJ mice with the gut microbiome composition and the benefits were transferable to non-healer mice via microbiome transplantation [20].

The enhanced cartilage repair in MRL/MpJ mice was also associated with the regulation of different signaling pathways such as phosphorylated Small Mothers Against Decapentaplegic 2/3 (pSMAD2/3), Wnt/β-catenin, pSMAD5, and decreases in metallopeptidase proteinase 9 (MMP9) and 13 (MMP13) [15], as well as enhanced extracellular vesicular function [21]. MRL/MpJ mice also demonstrated enhanced skeletal muscle healing via increased muscle regeneration and angiogenesis and reduced fibrosis and inflammation when compared to C57BL/6J mice [22]. Hypoxia-induced factor 1α (HIF1α) plays an important role in increasing the potency of muscle-derived stem cells as well as in enhancing muscle healing in MRL/MpJ mice [23].

More importantly, our previous study showed that MRL/MpJ mice possessed significantly higher bone microarchitecture and biomechanical properties at 2, 4, and 10 months of age when compared to both C57BL/10J and C57BL/6J mice. They also exhibited accelerated fracture healing and resistance to ovariectomy-induced bone loss in the 2 months following ovariectomy. Several signaling pathways were revealed, such as higher pSMAD5 expression during postnatal development as well as higher phosphorylated proteinase B (pAKT) and p38 mitogen-activated kinase (p38 MAPK) during injury, higher serum insulin-like growth factor (IGF1), and lower receptor activator of nuclear factor kappa-Β ligand (RANKL) in the serum [24].

The aim of this study was to investigate whether the MRL/MpJ mice resist age-related and long-term ovariectomy-induced bone loss when compared to WT mice, in order to identify potential mechanisms/factors to improve bone regeneration and repair after injury, disease, and aging.

## 2. Results

### 2.1. MRL/MpJ Mice Maintained Better Bone Microarchitecture at 14 Months Old (14 M) Compared to WT Mice

**Figure 1:**
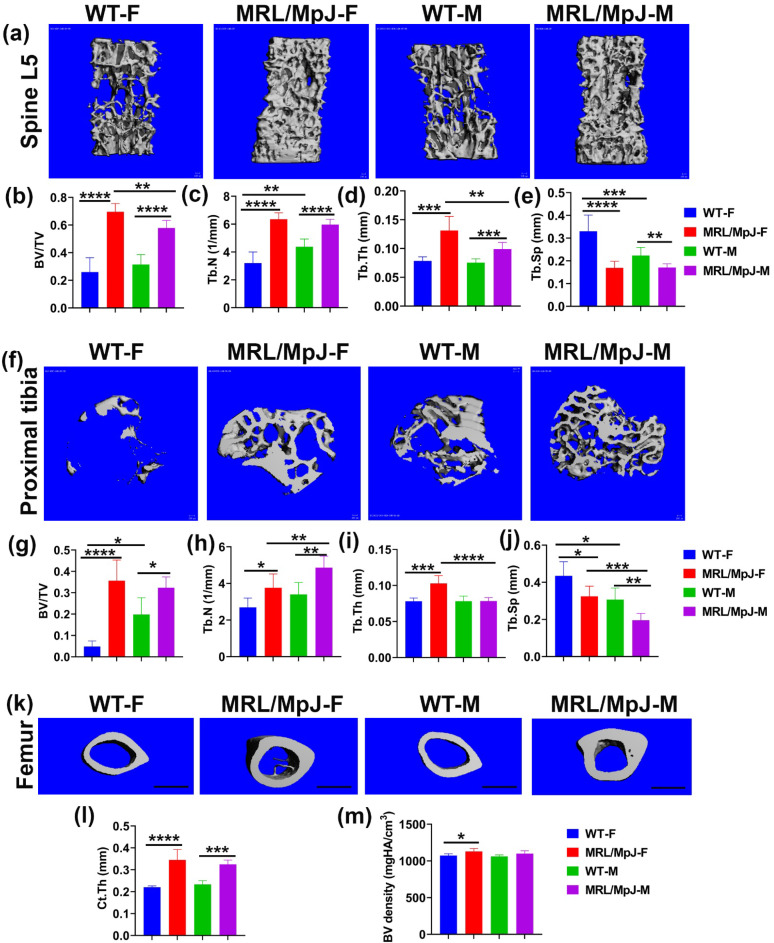
Micro-CT analysis for the lumbar spine, proximal tibia trabecular bone, and femur midshaft cortical bone of 14 M wild type (WT) and MRL/MpJ mice. (**a**) Micro-CT 3D view of spine L5 trabecular bone. MRL/MpJ-F and MRL/MPJ-M mice showed markedly higher trabecular bone mass than WT-F and WT-M. Scale bars = 100 μm. (**b**) BV/TV was significantly higher for MRL/MpJ-F and MRL/MpJ mice than WT-F mice and WT-M mice, respectively. The BV/TV of MRL/MpJ-F was significantly higher than MRL/MpJ-M. (**c**) The Tb.N of WT-M was significantly higher than WT-F. The Tb.N of MRL/MpJ-F and MRL/MpJ-M mice was significantly higher than that of WT-F (*p* < 0.0001) and MRL/MpJ-M, respectively. (**d**) The Tb.Th of MRL/MpJ-F and MRL/MpJ-M mice was significantly thicker than WT-F and WT-M mice, respectively. Furthermore, the Tb.Th of MRL/MpJ-F was significantly greater than MRL/MpJ-M. (**e**) The Tb.Sp of WT-M was significantly lower than WT-F mice (*p* = 0.012). The Tb.Sp of MRL/MpJ-F and MRL/MpJ-M mice was significantly lower than their WT counterparts. (**f**) Micro-CT 3D image of the proximal tibia (top view). More trabecular bone was observed in the WT-M mice than in the WT-F. MRL/MpJ-F and MRL/MpJ-M mice also displayed more trabecular bone than both WT-F and WT M. Scale bars = 100 μm. (**g**) BV/TV of the proximal tibia. The BV/TV of WT-M mice was significantly greater than WT-F mice. The BV/TV of MRL/MpJ-F mice and MRL/MpJ-M mice was significantly greater than the WT-F mice and WT-M mice, respectively. (**h**) Tb.N of the proximal tibia. The Tb.N of MRL/MpJ-F and MRL/MpJ-M mice was significantly greater than the WT-F and WT-M mice, respectively. A greater Tb.N was also observed in MRL/MpJ-M mice than in the MRL/MpJ-F mice. (**i**) Tb.Th of the proximal tibia. Higher Tb.Th was found in the MRL/MpJ-F and MRL-MpJ-M mice than in the WT-F and WT-M mice, respectively. Higher Tb.Th was also found in the MRL/MpJ-F mice than in the MRL/MpJ-M mice. (**j**) Tb.Sp of the proximal tibia. Lower Tb.Sp was observed in the WT-M mice than in the WT-F mice (*p* = 0.018). Lower Tb.Sp was also observed in the MRL/MpJ-M mice than in the MRL/MpJ-F mice. Lower Tb.Sp was also found in the MRL/MpJ-F and MRL/MpJ-M mice than the WT counterparts. (**k**) Micro-CT 3D view of midshaft femoral cortical bone. The cortical bone was thicker in the MRL/MpJ-F and MRL/MpJ-M mice than in the WT-F and WT-M mice, respectively. Trabecular bone was observed in the marrow cavity of the MRL/MpJ-F mice. Scale bars = 1 mm. (**l**) Cortical thickness of the femur midshaft. The Ct.Th of the MRL/MpJ-F and MRL/MpJ-M mice was significantly greater than the WT-F and WT-M mice, respectively. (**m**) The BV density of the MRL/MpJ-F mice was also significantly higher than the WT-F mice. * *p* < 0.05, ** *p* < 0.01,*** *p* < 0.001, **** *p* < 0.0001.

The micro-CT analysis of bone tissues of 14 M WT and MRL/MpJ mice was performed. The micro-CT 3D view of the spine L5 trabecular bone demonstrated markedly higher trabecular bone mass in both MRL/MpJ female (MRL/MpJ-F) and MRL/MpJ male (MRL/MpJ-M) mice than WT female (WT-F) and WT male (WT-M) mice (Figure 1a). The bone volume/total volume (BV/TV) of MRL/MpJ-F and MRL/MpJ-M mice was significantly higher than those of their WT counterparts (*p* < 0.0001 for both). MRL/MpJ-F mice also showed higher BV/TV than MRL/MpJ-M group mice (*p* < 0.01) (Figure 1b). The trabecular numbers (Tb.Ns) of MRL/MpJ-F and MRL/MpJ-M mice were significantly higher than those of their WT counterparts (*p* < 0.0001 for both). The Tb.N of WT-M was significantly higher than WT-F (*p* < 0.05) (Figure 1c). The trabecular thicknesses (Tb.Th) of MRL/MpJ-F and MRL/MpJ-M mice were also significantly thicker than those of their WT mice counterparts (*p* < 0.001 and *p* < 0.001 respectively). The Tb.Th of MRL/MpJ-F mice was significantly thicker compared to the MRL/MpJ-M mice (*p* < 0.01) (Figure 1d) as well. Furthermore, the trabecular separation (Tb.Sp) of MRL/MpJ-F and MRL/MpJ-M mice was significantly lower than that of WT-F (*p* < 0.0001) and WT-M (*p* < 0.01) mice, respectively. The Tb.Sp of WT-M mice was significantly lower when compared to WT-F mice (*p* = 0.01) (Figure 1e).

For the proximal tibia, 3D-reconstructed μCT images showed more trabecular bone in MRL/MpJ-F and MRL/MpJ-M mice than their WT counterparts (Figure 1f). More trabecular bone was also observed in the WT-M mice than in the WT-F mice. The BV/TV of MRL/MpJ-F and MRL/MpJ-M mice was dramatically higher than their WT counterparts (*p* < 0.0001; *p* < 0.05 respectively). The BV/TV of WT-M mice was significantly higher than WT-F mice (*p* < 0.05). No statistical differences were observed between MRL/MpJ-F and MRL-MpJ-M mice (Figure 1g). The Tb.N of MRL/MpJ-F and MRL/MpJ-M mice was significantly higher than those of WT-F (*p* < 0.05) and WT-M (*p* < 0.01), respectively. The Tb.N of MRL/MpJ-M mice was also significantly higher than MRL/MpJ-F mice (*p* = 0.01) (Figure 1h). Further, the Tb.Th of MRL/MpJ-F mice was significantly thicker than those of WT-F (*p* < 0.001) and MRL/MpJ-M mice (*p* < 0.0001), respectively (Figure 1i). The Tb.Sp of the MRL/MpJ-F and MRL/MpJ-M mice was markedly lower than that of WT-F mice (*p* < 0.05) and WT-M mice (*p* < 0.01), respectively. The Tb.Sp of WT-M and MRL/MpJ-M was significantly lower than that of WT-F mice (*p* < 0.05) and MRL/MpJ-F mice (*p* < 0.001), respectively (Figure 1j).

For the femoral cortical bone, 3D-reconstructed μCT images showed that the cortical bones were grossly thicker for MRL/MpJ-F and MRL/MpJ-M mice compared to their WT counterparts. Trabecular bone was observed in the marrow cavity of the female MRL/MpJ-F mice (Figure 1k). The cortical thickness (Ct.Th) of MRL/MpJ-F and MRL/MpJ-M mice was significantly thicker than those of WT-F mice (*p* < 0.0001) and WT-M mice (*p* < 0.001), respectively (Figure 1l). The BV density of MRL/MpJ-F mice was also significantly higher than that of WT-F mice (*p* < 0.05).

Taken together, both male and female MRL/MpJ mice demonstrated significantly better bone microarchitecture at an advanced age than their WT counterparts. Remarkably, MRL/MpJ-F mice showed similar and better bone parameters compared to the MRL/MpJ-M mice, which contrasts the differences seen between WT-F and WT-male mice.

### 2.2. MRL/MpJ Mice Demonstrated Significantly Greater Bone Matrix Architecture Compared to WT Mice at 14 M

**Figure 2:**
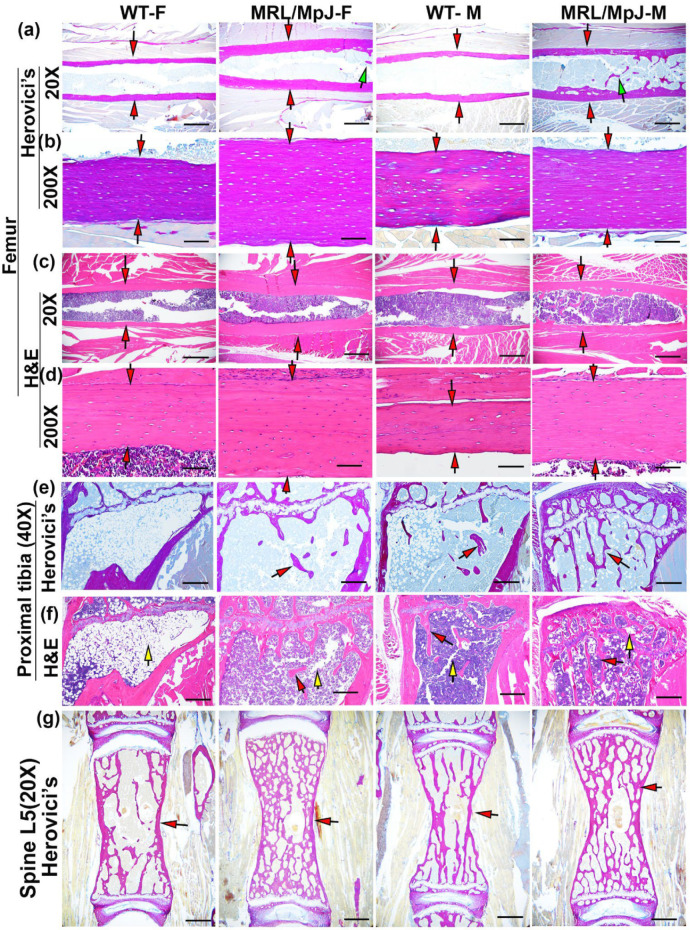
Histology of bone tissues of MRL/MpJ and WT mice at 14 M. (**a**) Herovici’s staining of femoral cortical bone at 20× magnification. Collagen type 1 (major bone matrix) stained pink-red and collagen type 3 stained dark blue. MRL/MJ-F and MRL/MpJ-M all showed thicker red cortical bone than the WT-F and WT-M mice, as demonstrated by red arrows. Note that trabecular bone was found in the MRL/MpJ-F and MRL/MJ-M midshaft femur marrow cavity, as indicated by green arrows. Scale bars = 1 mm. (**b**) Herovici’s staining of femoral cortical bone at 200× magnification. Highly organized COL1 was visible at 200× magnification in all groups. MRL/MJ-F and MRL/MpJ-M showed an obviously thicker COL1 bone matrix than their WT counterparts, indicated by red arrows. Scale bars = 100 μm. (**c**) H&E staining at 20× magnification, obviously thicker cortical bone was observed in MRL/MpJ-F and MRL/MpJ-M mice compared to the WT-F and WT-M mice, respectively, as demonstrated by red arrows. Scale bars = 1 mm. (**d**) H&E staining at 200× magnification. Relatively thicker cortical bone was observed for the MRL/MpJ-F and MRL/MpJ-M mice compared to the WT-M and WT-F mice, respectively, as indicated by red arrows. Scale bars = 100 μm. (**e**) Herovici’s staining of the proximal tibia below the growth plate at 40× magnification. WT-F mice showed almost no trabecular bone. WT-M mice showed sparse trabecular bone. MRL/MpJ-F mice showed the presence of trabecular bone, in red, but no bridging amongst trabeculae. MRL/MpJ-M mice showed relatively more dense trabecular bone with connections among trabecular bone. Red arrows demonstrate trabecular bone. Scale bars = 500 μm. (**f**) H&E staining of the proximal tibia at 40× magnification. WT-F mice showed almost no trabecular bone and a large number of adipose cells in the bone marrow region as indicated by yellow arrow. WT-M mice demonstrated little trabecular bone and scattered adipose cells (yellow arrow). Both MRL/MpJ-F and MRL/MpJ-M mice showed more trabecular bone than the WT-F and WT-M, respectively. Red arrows demonstrate trabecular bone; yellow arrows demonstrate adipose cells. Scale bars = 500 μm. (**g**) Herovici’s staining of spine L5 trabecular bones. MRL/MpJ-F and MRL/MpJ-M mice demonstrate remarkably denser trabecular bone than those of WT-F and WT-M, respectively, as demonstrated by red arrows. Scale bars = 1 mm.

Herovici’s staining was performed to differentiate collagen type 1 (COL1) and type 3 (COL3). COL1 (major bone matrix) stained pink-red and COL3 stained dark blue. For femoral cortical bone, MRL/MpJ-F and MRL/MpJ-M mice showed thicker pink-red cortical bone than their WT counterparts at 20× magnification (red arrows). Notably, trabecular bone (as demonstrated by green arrows) was found in the MRL/MpJ-F and MRL/MpJ-M mice marrow cavity of the midshaft of the femur, a location where trabecular bone is not normally present (Figure 2a). At 200× magnification, highly organized COL1-positive femoral cortical bone was visible in all groups (Figure 2b). MRL/MpJ-F and MRL/MpJ-M mice showed a thicker COL1 bone matrix than their WT counterparts (red arrows) (Figure 2b). H&E staining also demonstrated thicker femoral cortical bones in MRL/MpJ-F and MRL/MpJ-M mice than their WT counterparts, both at 20× magnification and 200× magnifications (red arrows) (Figure 2c,d).

For the proximal tibia, Herovici’s staining demonstrated almost no remaining trabecular bone in WT-F mice at 14 M while WT-M mice showed only sparse trabecular bone (red arrows). MRL/MpJ-F mice showed the presence of trabecular bone as pink-red COL1 in contrast to the absence of trabecular bone observed in the WT-F mice. MRL/MpJ-M mice showed relatively more dense trabecular bone in the proximal tibia (pink-red COL1) compared to the proximal tibia of WT-M mice as shown by red arrows (Figure 2e). H&E staining showed almost no trabecular bone but predominantly adipose cells occupying the bone marrow region of the WT-F mice (yellow arrow). WT-M mice showed a paucity of trabecular bone and scattered adipose cells (see yellow arrows), indicating bone loss. Both MRL/MpJ-F and MRL-MpJ-M mice showed more trabecular bone with only scattered adipose cells (yellow arrow) when compared to their WT counterparts (Figure 2f). For the spine L5, Herovici’s staining demonstrated more COL1-positive trabecular bone in the MRL/MpJ-F and MRL/MpJ-M mice compared to those of WT-F and WT-M mice, respectively, as indicated by red arrows (Figure 2g).

### 2.3. High Number of Osterix (OSX)^+^ Osteogenic Progenitor Cells with the Activation of pSMAD5, and Increased Osteoblasts Proliferation, Is Observed in the 14 M MRL/MpJ Mice Compared to WT Mice

**Figure 3:**
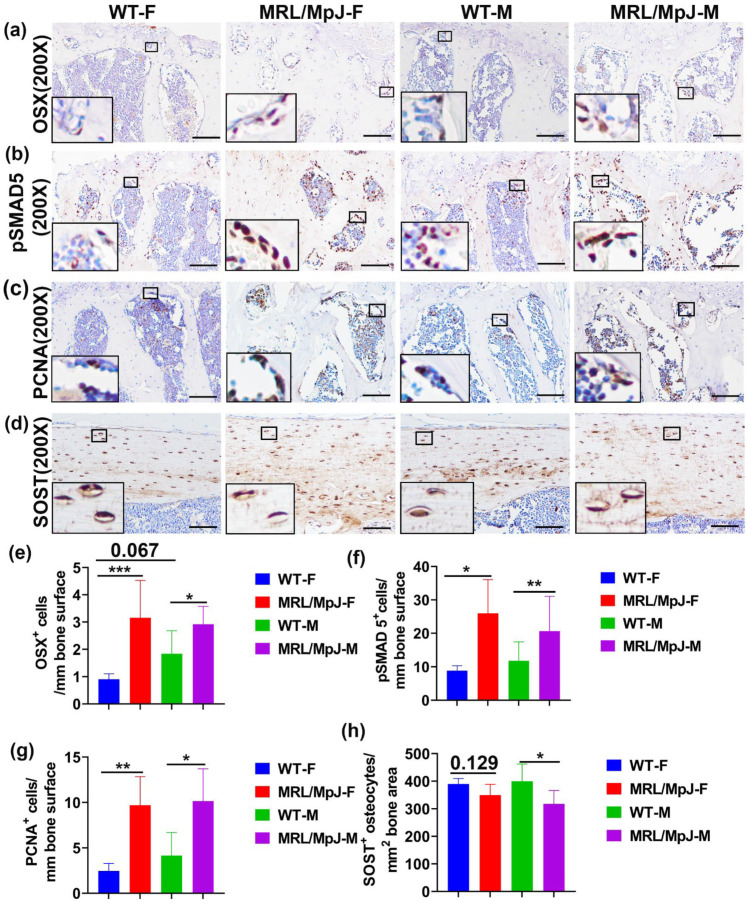
Immunohistochemical staining of 14 M bone tissues. (**a**,**e**) Immunohistochemistry staining of OSX in the L6 vertebra with quantification. OSX^+^ cells are stained brown (DAB) and nuclei are stained blue. Bone surface OSX^+^ cells in MRL/MpJ-F and MRL/MpJ-M mice were significantly greater than in WT-F and WT-M mice. There was a trend of increased bone surface OSX^+^ cell in WT-M mice compared to WT-F mice (*p* = 0.067). Scale bars = 100 μm. (**b**,**f**) Immunohistochemistry staining of the pSMAD5 of spine L6 trabecular bone with quantification. Positive cells are stained violet-red (Novared). Bone surface pSMAD5^+^ cell numbers were significantly higher in the MRL/MpJ-F and MRL/MpJ mice spine L6 trabecular bone than in the WT-F and WT-M mice, respectively. Scale bars = 100 μm. (**c**,**g**) Immunohistochemistry analysis of the PCNA of L6 vertebra trabecular bone with quantification. PCNA^+^ cells are stained brown (DAB). Bone surface PCNA^+^ cells were significantly greater in the trabecular bone of MRL/MpJ-F and MRL/MpJ-M mice than in the WT counterparts. Scale bars = 100 μm. (**d**,**h**) Immunohistochemistry of SOST of femoral cortical bone and quantification. SOST^+^ osteocytes and their lacuna-canalicular network are stained brown. SOST^+^ osteocytes in MRL/MpJ-F were relatively fewer than in WT-F mice (*p* = 0.129). SOST^+^ osteocytes in MRL/MpJ-M were significantly fewer than in WT-M mice. Scale bars = 100 μm. * *p* < 0.05, ** *p* < 0.01, *** *p* < 0.001.

Immunohistochemistry staining of OSX in the mouse lumbar spine tissues was performed. Images were taken for the entire L6 vertebrae (16–26 images based on spine size). Significantly more OSX^+^ cells on bone surface in MRL/MpJ-F and MRL/MpJ-M mice were observed when compared to WT-F mice (*p* < 0.01) and WT-M mice (*p* = 0.05), respectively. There was also a trend of increased OSX^+^ on bone surface in WT-M mice when compared to WT-F mice (*p* = 0.067) (Figure 3a,e). To investigate BMP-pSMAD5 signaling pathway activation, one of the most important signaling pathways for osteogenesis and bone formation [25], immunohistochemistry staining of pSMAD5 was performed. Positive cells are stained violet-red (Novared color reaction). The number of pSMAD5^+^ cells on the bone surface was significantly higher in the MRL/MpJ-F and MRL/MpJ-M mice than in the WT-F (*p* < 0.05) and WT-M mice (*p* = 0.01), respectively (Figure 3b,f). To investigate osteoblast proliferation, immunohistochemistry analysis of proliferating cell nuclear antigen (PCNA) was performed. PCNA is a cell proliferation marker with many interaction proteins related to DNA replication and repair [26]. PCNA-positive cells were stained as brown color (DAB color reaction). Bone surface PCNA^+^ cells were present in significantly higher quantities in the spine L5 trabecular bone of MRL/MpJ-F and MRL/MpJ-M mice than that of the WT-F (*p* < 0.01) and WT-M mice (*p* < 0.05), respectively (Figure 3c,g). Immunohistochemical staining of sclerostin (SOST) of femoral cortical bone was also performed. SOST^+^ osteocytes and their lacuna-canalicular network are stained brown. The number of SOST^+^ osteocytes in the femur of MRL/MpJ-F mice was relatively lower than that of the WT-F mice (*p* = 0.129). The number of SOST^+^ osteocytes in the femur of MRL/MpJ-M mice was significantly lower than that of WT-M mice (*p* < 0.05) (Figure 3d,h).

### 2.4. Serum Bone Marker Quantification in MRL/MpJ and WT Mice at 14 M

**Figure 4:**
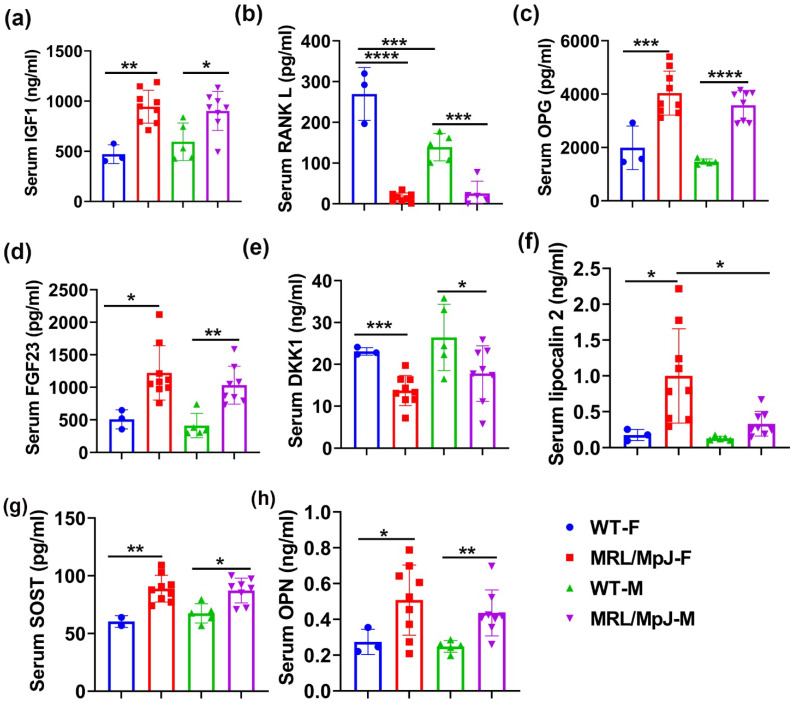
Serum bone-related marker levels of MRL/MpJ and WT mice at 14 M. (**a**) Serum IGF1 level. The IGF1 level was significantly higher in MRL/MpJ-F and MRL/MpJ-M mice than in WT-F and WT-M mice, respectively. (**b**) Serum RANKL level. The RANKL level in the serum was significantly higher in the WT-F mice than in the WT-M mice. RANKL was significantly lower in MRL/MpJ-F and MRL/MpJ-M mice than in the WT-F and WT-M mice, respectively. (**c**) Serum OPG level. OPG was significantly higher in the serum of MRL/MpJ-F mice than in their WT counterparts. (**d**) Serum FGF23 level. The FGF23 level was significantly increased in the serum of MRL/MpJ-F and MRL/MpJ-M mice compared to WT-F mice and WT-M mice, respectively. (**e**) Serum DKK1 level. The DKK1 level was significantly lower in MRL/MpJ-F and MRL/MpJ-M mice serum compared to that of the WT-F and WT-M mice serum, respectively. (**f**) Serum lipocalin 2 level. Lipocalin 2 was significantly higher in MRL/MpJ-F mice than the WT-F mice. Serum lipocalin 2 was also significantly higher in MRL/MpJ-F mice than in the MRL/MpJ-M mice. (**g**) Serum SOST level. The SOST level was significantly higher in MRL/MpJ-F and MRL/MpJ mice than in the WT-F and WT-M mice, respectively. (**h**) Serum OPN level. Serum OPN levels in MRL/MpJ-F and MRL/MpJ mice were significantly higher than in the WT-F and WT-M mice, respectively. All ANOVA: *p* < 0.0001. * *p* < 0.05, ** *p* < 0.01, *** *p* < 0.001, **** *p* < 0.0001.

Bone-metabolism-related proteins were measured using ELISA. The results demonstrated that IGF1 was significantly higher in MRL/MpJ-F mice than in WT-F mice (*p* < 0.01). IGF1 was significantly higher in the serum of MRL/MpJ-M mice than in the serum of WT-M mice (*p* < 0.01) (Figure 4a). RANKL is a critical ligand to promote osteoclast differentiation and its antibody has been approved by the Food and Drug Administration (FDA) of USA for treating osteoporosis [27,28,29,30,31]. RANKL in the serum was found at significantly higher levels in the WT-F mice than in the WT-M mice (*p* < 0.001). RANKL in the serum of MRL/MpJ-F mice was significantly lower than in WT-F mice (*p* < 0.0001). RANKL in the serum of MRL/MpJ-M mice was also significantly lower than in the serum of the WT-M mice (*p* < 0.001) (Figure 4b). No statistically significant differences were found for the serum levels of FGF21 or periostin between the groups.

Multiplex analyses using the Luminex system were also performed. Osteoprotegerin (OPG), a decoy receptor of RANKL, was significantly higher in the serum of MRL/MpJ-F mice than in WT-F (*p* < 0.001). The OPG level was also significantly higher in the serum of MRL/MpJ-M mice than in the serum of the WT-M mice (*p* < 0.0001) (Figure 4c). Fibroblast growth factor 23 (FGF23) is a bone-derived hormone that regulates blood phosphate by increasing its renal excretion and by decreasing the renal production of 1,25-dihydroxyvitamin D3 [32]. FGF23 was significantly increased in the sera of both MRL/MpJ-F and MRL/MpJ-M mice compared to WT-F mice (*p* < 0.05) and WT-M mice (*p* < 0.01), respectively. (Figure 4d). Dickkopf-1(DKK1) is a Wnt signaling inhibitor [33]. DKK1 was significantly lower in the serum of MRL/MpJ-F and MRL/MpJ-M mice than their WT counterparts (*p* = 0.001 and *p* < 0.05, respectively) (Figure 4e). Serum lipocalin 2 was significantly higher in MRL/MpJ-F mice than in WT-F mice (*p* < 0.05), as well as in MRL/MpJ-M mice (*p* < 0.05) (Figure 4f). SOST was significantly higher in the serum of MRL/MpJ-F mice than in the serum of WT-F mice (*p* = 0.01). The SOST level was also significantly higher in the serum of MRL/MpJ-M mice than in the serum of the WT-M mice (*p* < 0.05) (Figure 4g). Serum osteopontin (OPN) levels in both MRL/MpJ-F and MRL/MpJ-M mice were significantly higher than their WT counterparts (*p* < 0.05 and *p* < 0.01, respectively). No statistical differences between any groups for other factors measured were found.

### 2.5. MRL/MpJ Mice Demonstrated Less Severe Bone Loss after Long-Term Ovariectomy

**Figure 5:**
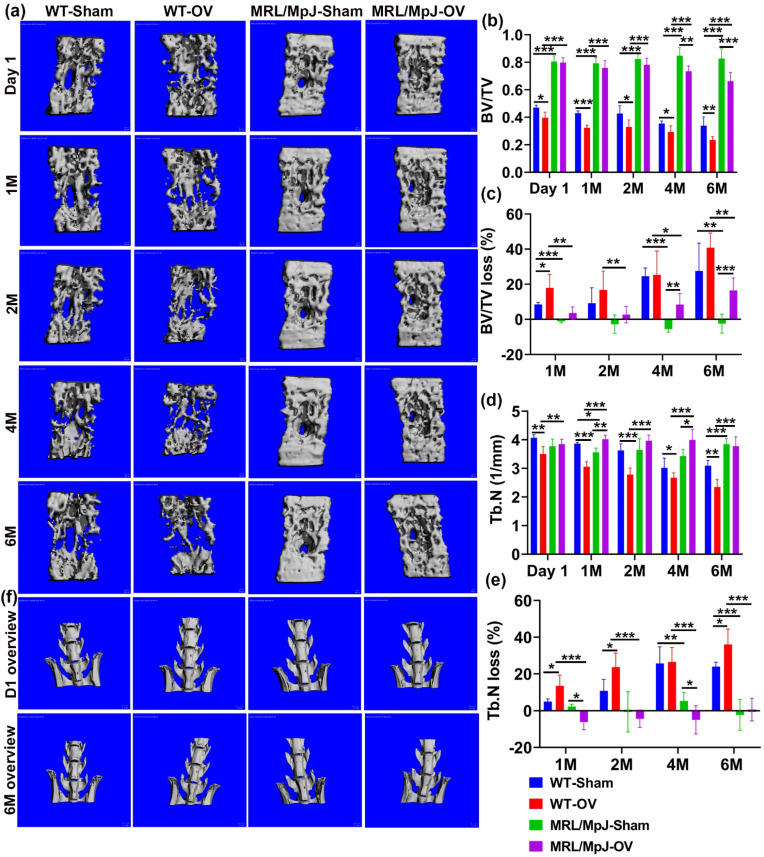
MicroCT analysis of spine L6 trabecular bone over the course of 6 M after ovariectomy. (**a**) Micro-CT 3D images of spine L6 at different timepoints after surgery. MRL/MpJ-Sham and MRL/MpJ-OV mice showed markedly higher trabecular bone mass than WT-Sham and WT-OV, respectively, at different timepoints after surgery. Scale bars = 100 μm (**b**) BV/TV. The BV/TV of the WT-OV mice progressively decreased over the 6 M period and was significantly lower than WT-Sham mice at day 1, 1 M, 2 M, 4 M, and 6 M. MRL/MpJ-OV mice showed significantly decreased BV/TV compared to MRL/MpJ-Sham mice only at 4 M and 6 M. MRL/MpJ-OV and MRL/MpJ-Sham mice showed significantly higher BV/TV than those of their WT counterparts, respectively, at all timepoints. (**c**) BV/TV loss percentage. Both WT-Sham and WT-OV mice showed BV/TV losses at 1 M, which continued to 6 M after surgery. MRL/MpJ-Sham mice did not show BV/TV losses (negative values) at any time during the 6 M period. MRL/MpJ-OV mice showed a significantly higher BV/TV loss percentage at 4 M and 6 M compared to MRL/MpJ-Sham mice. However, MRL/MpJ-OV mice showed a significantly lower BV/TV loss percentage at any timepoint when compared to WT-OV mice at 1 M, 2 M, 4 M, and 6 M. (**d**) Tb.N. WT-OV mice showed a lower Tb.N at day 1 than WT-Sham mice. MRL/MpJ-OV mice showed a significantly greater Tb.N than WT-OV at day 1. The Tb.N of WT-OV progressively decreased from 1 M to 6 M after surgery and was significantly less than the WT-Sham mice at 1 M, 2 M, and 6 M. In contrast, MRL/MpJ-OV showed increases in Tb.N at 1 M and 4 M after surgery compared to the MRL/MpJ-Sham mice and no difference at 6 M compared to MRL/MpJ-Sham mice. MRL/MpJ-OV mice showed a significantly greater Tb.N than WT-OV mice at all timepoints. (**e**) Tb.N loss percentage. WT-OV mice showed a significantly greater Tb.N loss percentage than WT-Sham at 1 M, 2 M, and 6 M, while MRL/MpJ-OV did not show Tb.N loss until 6 M and significantly less Tb.N loss at 1 M and 4 M than MRL/MpJ-Sham mice. MRL/MpJ-OV showed a significantly lower Tb.N loss percentage than WT-OV at all four timepoints. MRL/MpJ-Sham also showed significantly less Tb.N loss than WT-Sham at 4 M and 6 M after surgery. (**f**) Gross images of lumbar spine and hip at day 1 and 6 M after ovariectomy. WT-OV and WT-Sham mice showed iliac crest bone loss at 6 M compared to day 1 while MRL/MpJ-OV and MRL/MpJ-Sham did not show obvious bone loss. Scale bars = 1 mm. * *p* < 0.05, ** *p* < 0.01, *** *p* < 0.001.

In vivo micro-CT scanning was performed for the lumbar spine and proximal tibia trabecular bone after ovariectomy surgery. For the spine L5 trabecular bone, micro-CT 3D images demonstrated a higher trabecular bone mass in MRL/MpJ-Sham and MRL/MpJ-OV mice compared to the WT counterparts, at various timepoints after ovariectomy surgery (Figure 5a). Quantification analysis showed progressive decreases in the BV/TV in WT-OV mice over the 6-month (6 M) period and significantly lower BV/TV than WT-Sham mice at 1 M, 2 M, 4 M, and 6 M. In contrast, MRL/MpJ-OV showed significant decreases in BV/TV compared to the MRL/MpJ-Sham mice at only 4 M and 6 M (*p* < 0.01 and 0.001, respectively). MRL/MpJ-Sham and MRL/MpJ-OV mice showed significantly higher BV/TV than their WT counterpart mice at all timepoints (All *p* < 0.0001, Figure 5b). Since MRL/MpJ mice have higher bone BV/TV than WT mice, BV/TV loss percentage was calculated to accurately reflect the resistance of MRL/MpJ mice to ovariectomy-induced bone loss. The BV/TV loss percentage of each group was calculated relative to each of its day 1 values. The results indicate that both WT-Sham and WT-OV mice showed BV/TV loss at 1 M, continuing until 6 M after surgery, as indicated by positive bone loss percentage. There was significantly more BV/TV loss percentage in WT-OV mice than WT-Sham mice at 1 M (*p* < 0.05). There were no significant differences between these two groups for other timepoints. In contrast, the MRL/MpJ-Sham mice did not show BV/TV loss at any time during the 6 M period after the sham surgery. In fact, the negative bone loss percentage values reflect bone gain. MRL/MpJ-OV mice showed significantly more BV/TV loss percentage at 4 M and 6 M after ovariectomy surgery when compared to the MRL/MpJ-sham mice (*p* < 0.01 and *p* < 0.001, respectively) (Figure 5c). However, MRL/MpJ-OV mice showed significantly less BV/TV loss percentage at all timepoints when compared to the WT-OV counterparts (Figure 5c). MRL/MpJ-Sham mice also showed significantly less BV/TV loss percentage than that of WT-Sham mice at 1 M, 4 M, and 6 M (*p* < 0.001, *p* < 0.001, *p* < 0.01, respectively).

The Tb.N of WT-OV mice progressively decreased from 1 M to 6 M after surgery and to a level that was significantly lower than the WT-Sham group at all timepoints. In contrast, the Tb.N of MRL/MpJ-OV mice increased over time and was significantly greater at 1 M, 2 M, and 4 M after surgery compared to MRL/MpJ-Sham mice, which showed no difference at 6 M. MRL/MpJ-OV mice showed a significantly higher Tb.N than WT-OV mice at all timepoints (*p* < 0.001 for all timepoints). MRL/MpJ Sham mice also showed a significantly higher Tb.N than the WT-Sham mice at 6 M after surgery (*p* < 0.001) (Figure 5d). The Tb.N loss percentage relative to day 1 was then calculated for all groups. WT-OV mice showed a significantly higher Tb.N loss percentage than the WT-Sham mice at 1 M, 2 M, and 6 M. In contrast, the MRL/MpJ-OV mice did not show a Tb.N loss until 6 M (all Tb.N loss percentages are negative values, except 6 M). A smaller Tb.N loss percentage at 1 M and 4 M was observed in the MRL/MpJ-OV mice compared to the MRL/MpJ-Sham mice. Strikingly, MRL/MpJ-OV mice displayed a significantly lower Tb.N loss percentage when compared to the WT-OV group at all four timepoints (*p* < 0.001 for all timepoints). The MRL/MpJ-Sham mice also displayed a significantly lower Tb.N loss percentage when compared to WT-Sham mice at 4 M and 6 M after surgery (*p* < 0.01 and 0.001, respectively) (Figure 5e).

Tb.Th changes after ovariectomy were further examined in both WT and MRL/MpJ mice. The Tb.Th of the spine L6 of MRL/MpJ mice with and without OV was consistently thicker than the WT mice (sham or OV) (all *p* values are <0.001). At 1 M, 2 M, and 6 M after ovariectomy, WT-OV mice showed significant decreases in Tb.Th compared to the WT-Sham mice (*p* < 0.001, 0.05, and 0.01, respectively). In comparison, the MRL/MpJ-OV mice only demonstrated significant decreases in Tb.Th at 4 M and 6 M compared to the MRL/MpJ-Sham mice (Supplemental Figure S1a). The Tb.Th loss percentage of the L6 vertebra was also calculated and compared among the groups. At 1 M, both the MRL/MpJ-OV and WT-OV mice showed a significantly greater Tb.Th loss percentage than their sham counterparts (both *p* < 0.05). At 4 M, MRL/MpJ-OV mice also showed significantly greater Tb.Th loss percentage than the MRL/MpJ-Sham mice and the WT OV mice (*p* < 0.01 and *p* < 0.05, respectively). However, MRL/MpJ-Sham mice did not show Tb.Th loss and showed a significantly lower Tb.Th loss percentage than the WT-Sham mice. At 6 M, the MRL/MpJ-OV mice showed a significantly higher Tb.Th loss percentage than the MRL/MpJ-Sham mice. No statistical differences were found among other groups. These results indicate that Tb.Th loss contributes to bone loss in both the WT and MRL/MpJ mice after ovariectomy (Supplemental Figure S1b).

In addition, changes in Tb.Sp were also compared among the groups. WT-OV mice showed a significant increase in Tb.Sp at day 1, 1 M, 2 M, and 6 M compared to WT-Sham mice (*p* < 0.05, 0.001, 0.001, and 0.05, respectively). In contrast, MRL/MpJ-OV mice did not show significant Tb.Sp increases at any timepoints after surgery compared to MRL/MpJ-Sham mice. The MRL/MpJ-OV mice showed significantly less Tb.Sp compared to WT-OV mice at 1, 2, 4, and 6 M after surgery (*p* < 0.05, 0.01, 0.01, and 0.001, respectively) (Supplemental Figure S1c). The Tb.Sp increase percentage was then calculated and compared among the groups. The Tb.Sp increase percentages of the WT-OV mice were significantly greater at 1 M and 2 M than the WT-Sham mice (*p* < 0.05 for both), while no significant differences were found at 4 M and 6 M. The Tb.Sp increase percentage in the MRL/MpJ-OV mice did not show a significant difference at any timepoint when compared to the MRL/MpJ-Sham mice. The MRL/MPJ-OV mice showed significantly lower Tb.Sp increase percentages than the WT-OV mice at 1 M, 2 M, 4 M, and 6 M (All *p* values < 0.01). MRL/MpJ-Sham mice also demonstrated significantly smaller Tb.Sp increase percentages at 2 M, 4 M, and 6 M compared to the WT-Sham mice (Supplementary Figure S1d).

Furthermore, gross images of the lumbar spine and hip were observed at day 1 and 6 months after ovariectomy. Both WT-OV and WT-Sham mice showed iliac crest bone loss at 6 months compared to day 1 (Figure 5f), while MRL/MpJ mice with or without OV did not show obvious bone loss (Figure 5g). Taken together, MRL/MpJ-OV mice showed a significant reduction in BV/TV loss percentage of spine L6, which is likely attributed to a smaller Tb.N loss percentage and Tb.Sp increase percentage. The MRL/MpJ-OV mice showed a similar Tb.Th loss percentage to the WT-OV mice. These results indicate that estrogen may not play a significant role in maintaining higher bone mass in MRL/MpJ mice.

**Figure 6:**
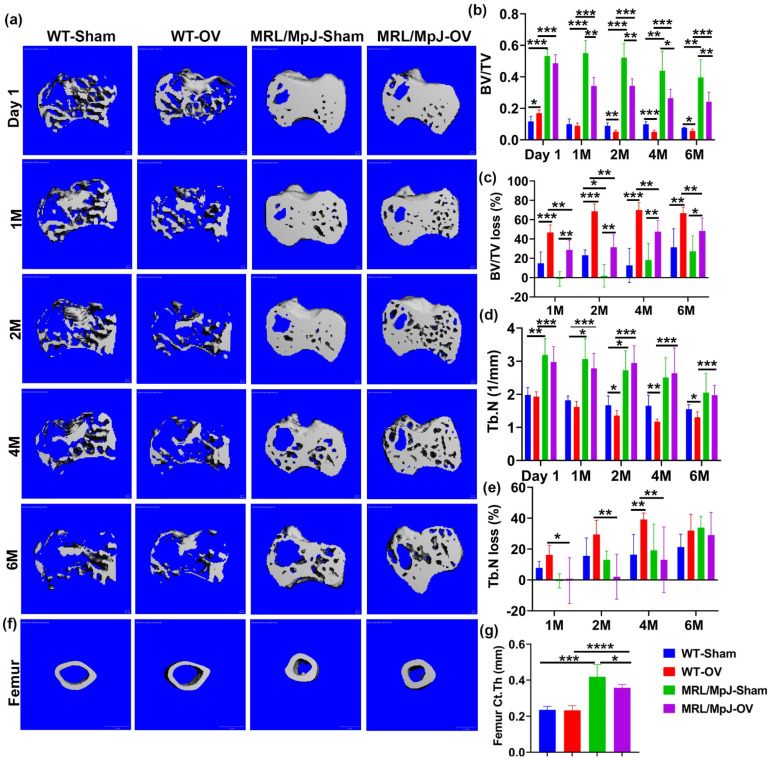
Micro-CT analysis of proximal tibia trabecular bone and femoral cortical bone. (**a**) Micro-CT 3D images of the proximal tibia over the course of 6 months. WT-OV mice demonstrated obvious trabecular bone loss at 1 month compared to day 1 after surgery and continued bone loss until 6 months, at which point almost no trabecular bone remained. WT-Sham mice also demonstrated bone loss at 6 M. MRL/MpJ-OV also demonstrated bone loss during the 6 M period after ovariectomy but to a much lesser extent. Markedly higher bone mass was maintained during this process for both MRL/MpJ-OV and MRL/MpJ-Sham. Scale bars = 100 μm. (**b**) BV/TV. At day 1, BV/TV was significantly higher in WT-OV than WT-Sham mice. At 2 M to 6 M, the BV/TV in the WT-OV group was significantly lower than that of the WT-Sham group. The MRL/MpJ-OV group demonstrated decreased BV/TV when compared to the MRL/MpJ-Sham group at the 1 M, 2 M, 4 M, and 6 M timepoints. However, MRL/MpJ-OV and MRL/MpJ-Sham mice maintained significantly higher BV/TV than the WT-OV and WT-Sham groups at all timepoints. (**c**) BV/TV loss percentage relative to day 1. At 1 M, WT-OV mice lost 45% of BV/TV while MRL/MpJ-OV lost 25%. At 2 M, WT-OV lost 70% of BV/TV while MRL/MPJ-OV mice lost 25% of BV/TV. At 4 M and 6 M, WT-OV maintained 70% bone loss while MRL/MpJ-OV mice maintained 40% bone loss and did not further progress from 4 M to 6 M. WT-OV and MRL/MpJ-OV both showed significantly higher BV/TV than WT-Sham and MRL/MpJ-Sham at all timepoints, respectively. MRL/MpJ-OV showed a significantly lower BV/TV loss percentage compared to WT-OV at all timepoints. (**d**) Tb.N of the proximal tibia. MRL/MpJ-OV mice showed a significantly higher Tb.N at day 1 and 1, 2, 4, and 6 M than their WT-OV counterparts. MRL/MpJ-Sham also showed a significantly higher Tb.N than the WT-Sham mice at day 1, 1 M, and 2 M, respectively. WT-OV mice showed a significantly decreased Tb.N compared to WT-Sham at 2 M, 4 M, and 6 M. MRL/MpJ-OV mice did not show significant differences compared to MRL/MpJ-Sham mice at any timepoint. (**e**) Tb.N loss percentage relative to day 1. At 1 M, 2 M, and 4 M, MRL/MpJ-OV mice demonstrated a significantly smaller Tb.N loss percentage than WT-OV mice. WT-OV mice lost 40% of their Tb.N and showed a significantly greater Tb.N loss percentage than WT-Sham at 4 M. No difference was found at 6 M after ovariectomy among any groups. (**f**) Micro-CT 3D images of femoral cortical bone at 6 M after surgery. Both MRL/MpJ-Sham and MRL/MpJ-OV mice maintained thicker femoral cortical bone compared to their WT counterparts. Scale bars = 1 mm. (**g**) Ct.Th at 6 M after ovariectomy. MRL/MpJ-Sham and MRL/MpJ-OV showed significantly greater Ct.Th than WT-Sham and WT-OV, respectively. MRL/MpJ-OV mice also exhibited decreases in Ct.Th compared to MRL/MpJ-Sham mice. * *p* < 0.05, ** *p* < 0.01, *** *p* < 0.001. **** *p* < 0.0001.

For the proximal tibia, 3D-reconstructed μCT images of WT-OV mice showed obvious trabecular bone loss at 1 M compared to day 1 after surgery with continuous bone loss until 6 months, at which point almost no trabecular bone remained. WT-Sham mice also demonstrated bone loss at 6 M. The MRL/MpJ-OV also showed bone loss during the 6 M period following ovariectomy but to a much lesser extent. Markedly higher bone mass was maintained during this process for both the MRL/MpJ-OV and MRL/MpJ-Sham mice (Figure 6a). At day 1, BV/TV was significantly higher in WT-OV mice when compared to WT-Sham mice but significantly lower at 2 M, 4 M, and 6 M after surgery. At 1 M, the WT-OV mice did not show significant decreases in BV/TV compared to the WT-Sham group, likely due to its initially higher BV/TV than WT-Sham mice. The initially higher BV/TV in the WT-OV mice was likely associated with individual animal variation. MRL/MpJ-OV mice also showed significantly lower BV/TV at 1 M, 2 M, 4 M, and 6 M compared to MRL/MpJ-Sham mice (*p* < 0.01, *p* < 0.01, *p* < 0.05, and *p* < 0.05, respectively). Both MRL/MpJ-Sham and MRL/MpJ-OV showed significantly higher BV/TV than their WT counterparts at all timepoints. (Figure 6b). Furthermore, BV/TV loss percentage was calculated as compared to day 1. At 1 M, the WT-OV mice lost 45% of their BV/TV, while the MRL/MpJ-OV mice lost 25%. The WT-OV and MRL/MpJ-OV mice both showed a significantly higher BV/TV loss percentage than WT-Sham and MRL/MpJ-Sham mice, respectively (*p* < 0.001 and *p* < 0.01, respectively). The MRL/MpJ-OV mice showed a significant reduction in BV/TV loss percentage compared to WT-OV (*p* < 0.01). At 2 M, the WT-OV mice lost 70% of BV/TV while the MRL/MpJ-OV mice lost 25% of BV/TV. The MRL/MpJ-OV mice showed a significantly lower BV/TV loss percentage than the WT-OV mice (*p* < 0.01). The MRL/MpJ-Sham mice demonstrated a significantly lower BV/TV loss percentage than the WT-Sham mice (*p* < 0.05). At 4 M and 6 M, the WT-OV group maintained 70% bone loss while the MRL/MpJ-OV mice maintained 40% bone loss and did not progress further from 4 M to 6 M. The BV/TV loss percentage was significantly lower in the MRL/MpJ-OV mice compared to the WT-OV mice (*p* < 0.01 for both timepoints) (Figure 5c).

For Tb.N quantification, the MRL/MpJ-Sham mice showed a significantly greater Tb.N compared to WT-Sham mice at day 1, 1 M, and 2 M after surgery (*p* < 0.01, *p* < 0.05, *p* < 0.05, respectively). MRL/MpJ-OV mice also showed a significantly higher Tb.N at day 1 as well as 1, 2, 4, and 6 M after ovariectomy as compared to their WT-OV counterparts (all *p* < 0.001). The WT-OV group showed significant Tb.N decreases at 2, 4, and 6 M compared to the WT-Sham group (*p* < 0.05, *p* < 0.01, and *p* < 0.05, respectively). The MRL/MpJ-OV mice did not show significant Tb.N decreases compared to the MRL/MpJ-Sham mice at any timepoints (Figure 6d). Tb.N loss percentages of all groups were also calculated relative to day 1. The MRL/MpJ-OV mice demonstrated a significantly lower Tb.N loss percentage at 1 M, 2 M, and 4 M compared to WT-OV mice (*p* < 0.05, *p* < 0.01, *p* < 0.01, respectively. WT-OV mice also showed a significantly higher Tb.N loss percentage at 4 M than WT-Sham mice after surgery (*p* < 0.01). No difference was found at 6 M after ovariectomy among any groups (Figure 6e).

Tb.Th changes in the proximal tibia were compared at different timepoints after ovariectomy. MRL/MpJ-Sham and MRL/MPJ-OV mice maintained significantly higher Tb.Th than their WT counterparts, at all timepoints, respectively (all statistically significant). Both WT-OV and MRL/MpJ-OV mice showed significant decreases in Tb.Th compared to their sham counterpart mice at 2, 4, and 6 M after ovariectomy surgery (all statistically significant) (Supplemental Figure S1e). Furthermore, the Tb.Th loss percentage was also calculated and compared among the groups. Neither WT-Sham nor MRL/MpJ-Sham mice showed a significantly greater Tb.Th loss percentage at any timepoints (all loss percentages are negative values). Both WT-OV and MRL/MpJ-OV mice showed a significantly greater Tb.Th loss percentage than their sham counterpart mice at 2 M, 4 M, and 6 M post-surgery (all statistically significant) (Supplemental Figure S1f). These results indicated that ovariectomy also caused Tb.Th loss in MRL/MpJ mice.

Additionally, Tb.Sp changes in the proximal tibia were also measured and compared among the different groups. The MRL/MpJ-Sham and MRL/MpJ-OV mice showed significantly lower Tb.Sp at day 1 than their WT counterparts (*p* < 0.05 and *p* < 0.001, respectively). WT-OV mice showed significantly higher Tb.Sp at 4 M and 6 M compared to that of WT-Sham mice (*p* < 0.01 and *p* < 0.05, respectively). MRL/MpJ-OV mice showed a significant reduction in Tb.Sp when compared to WT-OV mice at 1 M, 2 M, 4 M, and 6 M after surgery (Supplemental Figure S1g). Finally, the Tb.Sp increase percentage was significantly higher in WT-OV mice than the WT-Sham mice (*p* < 0.01) at 4 M after surgery. MRL/MpJ-OV mice did not show a significantly higher Tb.Sp increase percentage when compared to the MRL/MpJ-Sham mice at any timepoint. However, the MRL/MpJ-OV mice demonstrated significant reductions in the Tb.Sp increase percentage compared to the WT-OV at 1 M, 2 M, 4 M, and 6 M after surgery (all are statistically significant) (Supplemental Figure S1h).

Femoral cortical bone parameters were further assessed and compared among the groups. Three-dimensional reconstructed μCT images at 6 M after ovariectomy demonstrated thicker cortical bone for the MRL/MpJ-OV and MRL/MpJ-Sham mice than the WT-OV and WT-Sham mice, respectively (Figure 6f). The quantification of Ct.Th indicated that the MRL/MpJ-Sham and MRL/MpJ-OV mice had significantly higher Ct.Th than their WT counterparts (*p* < 0.001 and 0.0001). However, the MRL/MpJ-OV mice also showed a significantly decreased Ct.Th compared to the MRL/MpJ-Sham mice (*p* < 0.05). No statistical difference for Ct.Th between WT-OV and WT-Sham mice was found (Figure 6g). The femoral cortical bone density showed no statistical difference between any groups.

### 2.6. Histology of Bone Tissues 6 M Following Ovariectomy

**Figure 7:**
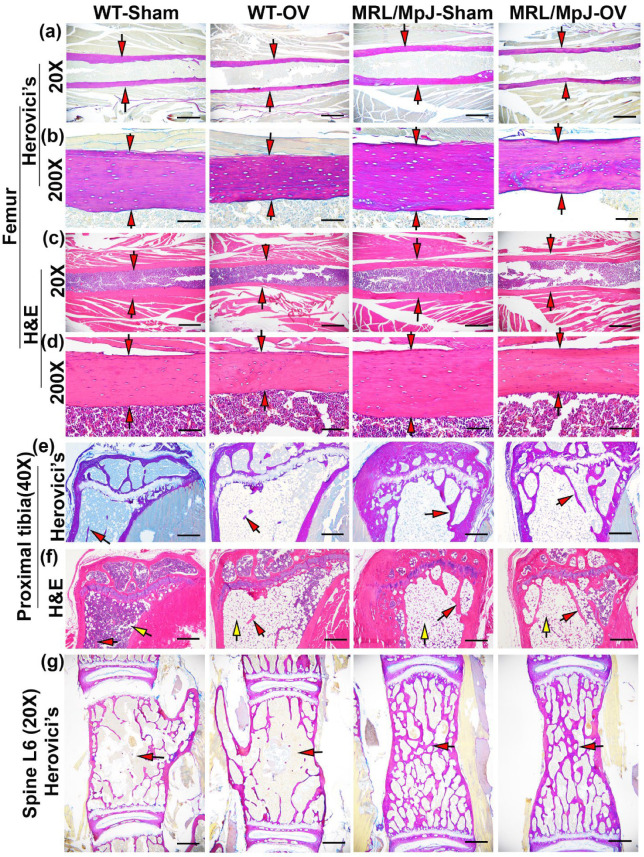
Histology at 6 M after ovariectomy or sham surgery. (**a**,**b**) Herovici’s staining of femoral cortical bone at 20× and 200× magnification. At 20× magnification, both WT-OV and MRL/MpJ-OV showed a relatively thinner collagen type 1 (COL1) matrix compared to WT-Sham and MRL/MpJ-Sham, respectively (pink-red), as indicated by red arrows. Scale bars = 1 mm. At 200× magnification, all groups showed a highly organized pink-red COL1 matrix. WT-OV and MRL/MpJ-OV demonstrated obviously thinner cortical bone than WT-Sham and MRL/MpJ-Sham, respectively, as demonstrated by red arrows. Scale bar = 100 μm. (**c**,**d**) H&E staining of femoral cortical bone at 20× and 200×. At 20×, WT-OV and MRL/MpJ-OV mice both exhibited relatively thinner femoral cortical bone compared to the WT-Sham and MRL/MpJ-Sham groups as indicated by red arrows, respectively. Scale bars = 1 mm. At 200× magnification. Both WT-OV and MRL/MpJ-OV showed obviously thinner femoral cortical bone, as indicated by red arrows. Osteocytes were well-organized. Scale bars = 100 μm. (**e**) Herovici’s staining of proximal tibia trabecular bone. WT-OV mice lost almost all pink-red-stained COL1-positive trabecular bone, while WT-Sham mice only had minimally pink-stained trabecular bone remaining, as shown by red arrows. In contrast, MRL/MpJ-OV maintained more trabecular bone, which was similar to MRL/MpJ-Sham mice. Scale bars = 500 μm. (**f**) H&E staining of the proximal tibia. WT-OV mice showed almost no trabecular bone and large amounts of adipose tissue (empty space with flat nuclei) occupying the bone marrow cavity compared to scattered adipose cells in the WT-Sham mice. MRL/MpJ-OV and MRL/MpJ-Sham both showed more trabecular bone than WT-Sham and WT-OV mice but large amounts of adipose tissue were also observed in both MRL/MpJ-Sham and MRL/MpJ-OV mice. Red arrows indicate trabecular bone while yellow arrows indicate fat cells. Scale bars = 500 μm. (**g**) Herovici’s staining of spine L6. The pink-red-stained trabecular bones in WT-OV mice were slightly more sparse and thinner than the WT-Sham. On the contrary, MRL/MpJ-Sham and MRL/MpJ-OV showed significantly denser pink-red-stained COL1-positive trabecular bone than WT-Sham and WT-OV with no obvious differences between the MRL/MpJ-OV and MRL/MpJ-Sham groups. Red arrows indicate trabecular bones. Scale bars = 1 mm.

Herovici’s staining was performed for bone tissues of the mice undergoing ovariectomy and sham surgery at 6 M. At 20× magnification, both the WT-OV and MRL/MpJ-OV groups had a thinner COL1 matrix compared to the WT-Sham and MRL/MpJ-Sham groups, respectively, in femoral cortical bone is indicated by red arrows (pink-red) (Figure 7a). At 200× magnification all groups showed a highly organized COL1 matrix in femoral cortical bone (pink-red), while the WT-OV and MRL/MpJ-OV mice showed thinner cortical bone compared to the WT-Sham and MRL/MpJ-Sham group. However, the MRL/MpJ-Sham and MRL/MpJ-OV mice showed relatively thicker cortical bone when compared to the WT-Sham and WT-OV groups, respectively (Figure 7b). H&E staining results showed that at 20× magnification, the WT-OV and MRL/MpJ-OV mice both showed relatively thinner femoral cortical bone as indicated by red arrows, which correlates with the micro-CT results (Figure 7c). At 200× magnification, both the WT-OV and MRL/MpJ-OV cohorts had thinner femoral cortical bone compared to the WT-Sham and MRL/MpJ-Sham cohorts. In all groups, osteocytes were well-organized and there were no pathological changes observed in any group (Figure 7d).

For the proximal tibia trabecular bone, Herovici’s staining demonstrated that WT-OV mice lost almost all pink-stained trabecular bone, while the WT-Sham mice also had only minimal pink-stained trabecular bone. In contrast, the MRL/MpJ-OV maintained substantial trabecular bone in a similar manner as the MRL/MpJ-Sham mice (Figure 7e). H&E staining revealed an almost complete absence of trabecular bone with large amounts of adipose tissue (empty space with flat nuclei pointed by yellow arrows) in the bone marrow cavity of the WT-OV mice with just scattered adipose cells in the WT-Sham mice. The MRL/MpJ-Sham and MRL/MPJ-OV mice both showed more trabecular bone than their WT counterparts, respectively. However, large amounts of adipose tissue (pointed by yellow arrows) were also found in the MRL/MpJ-Sham and MRL/MpJ-OV mice (Figure 7f). Furthermore, Herovici’s staining showed that the pink-red-stained trabecular bone of spine L6 in WT-OV mice was sparse and thinner than in the WT-Sham mice. In contrast, the MRL/MpJ-Sham and MRL/MpJ-OV mice showed significantly thicker and denser pink-red-stained COL1 trabecular bone than their WT counterparts. No obvious difference was found between MRL/MpJ-OV and MRL/MpJ-Sham mice (Figure 7g).

### 2.7. MRL/MpJ Mice Maintained Higher OSX^+^ Cells, High pSMAD5^+^ and PCNA^+^ Cells on Bone Surface, and Lower TRAP^+^ Osteoclasts after Ovariectomy

**Figure 8:**
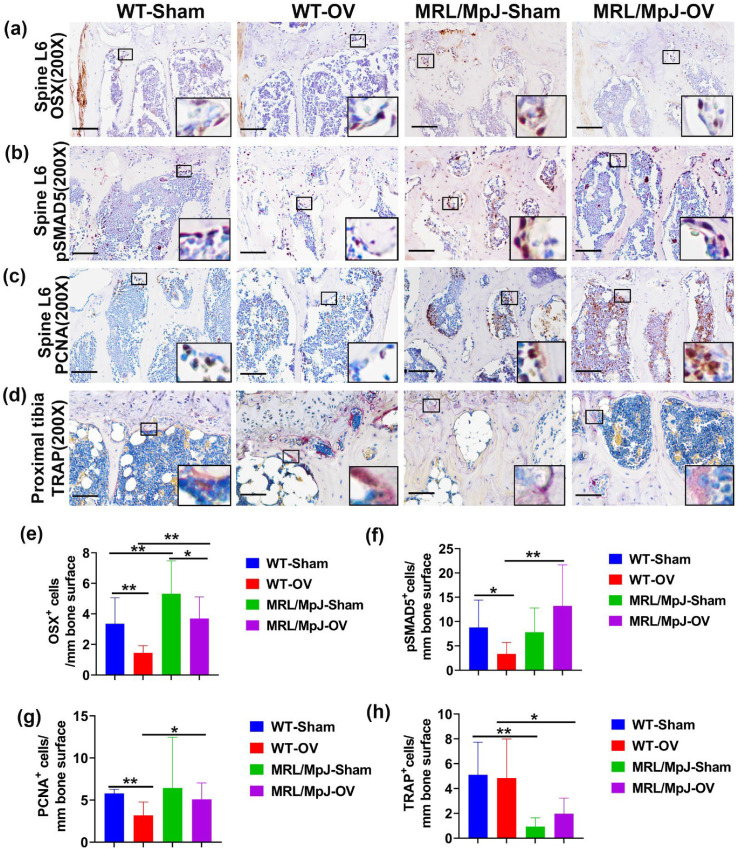
Immunohistochemistry and TRAP staining at 6 M after ovariectomy. (**a**,**e**) Immunohistochemical staining of OSX and quantification. OSX^+^ cells are stained brown. Nuclei are stained blue. Insets highlight positive cells in each group. Scale bars = 100 μm. The number of OSX^+^ cells on the bone surface of WT-OV and MRL/MpJ-OV mice was significantly lower than the WT-Sham mice or MRL/MpJ-Sham mice. However, MRL/MpJ-OV and MRL/MpJ-Sham mice maintained significantly higher numbers of OSX^+^ cells on the bone surface compared to WT-OV and WT-Sham, respectively. (**b**,**f**) pSMAD5 staining and quantification. pSMAD5^+^ cells are stained violet-red (Novared). Insets demonstrate pSMAD5^+^ cells on the bone surface. Scale bars = 100 μm. The pSMAD5^+^ cells on the bone surface were decreased in WT-OV mice as compared to the WT-Sham. MRL/MpJ-OV mice did not show decreases in pSMAD5^+^ compared to MRL/MpJ-Sham mice. MRL/MpJ-OV mice showed significantly greater numbers of pSMAD5^+^ cells compared to WT-OV mice. (**c**,**g**) PCNA staining and quantification. PCNA^+^ cells are shown in brown. PCNA^+^ cells are located both on the bone surface and in the bone marrow. Insets highlight PCNA^+^ cells. Scale bars = 100 μm. The number of PCNA^+^ cells on the bone surface decreased in the WT-OV group compared to the WT-Sham group. The MRL/MpJ-OV group showed a significantly greater number of PCNA^+^ cells than the WT-OV mice. (**d**,**h**) TRAP staining for osteoclast and quantification in the proximal tibia. TRAP^+^ cells are stained violet-red on the bone surface with single or multiple nuclei. Insets highlight TRAP^+^ cells. Scale bars = 100 μm. Both WT-OV and MRL/MPJ-OV mice did not show significant changes in TRAP^+^ cells compared to WT-Sham or MRL/MpJ-Sham mice. However, MRL/MpJ-Sham and MRL/MpJ-OV mice both showed significantly lower TRAP^+^ cells on the bone surface than WT-Sham and WT-OV, respectively (*p* = 0.006 and 0.047, respectively). * *p* < 0.05, ** *p* < 0.01.

Immunohistochemistry staining of spine L6 trabecular bone showed OSX^+^ cells on the bone surface as brown nuclei staining. The OSX^+^ cell number on the bone surface was significantly lower in the WT-OV mice than in the WT-Sham mice (*p* = 0.01). The OSX^+^ cell number was also significantly lower in the MRL/MpJ-OV mice compared to the MRL/MpJ-Sham mice (*p* = 0.05). However, the MRL/MPJ-Sham and MRL/MpJ-OV mice maintained significantly higher numbers of OSX^+^ cells on the bone surface compared to their WT counterparts (*p* = 0.005 and 0.01, respectively) (Figure 8a,e).

Immunohistochemistry staining of pSMAD5^+^ of the spine L6 trabecular bone showed violet-red-positive cells (Novared color reaction) on bone surface osteoblasts. The pSMAD5^+^ cells on the bone surface were found to be decreased in the WT-OV mice when compared to the WT-Sham mice (*p* < 0.05). The MRL/MpJ-OV mice did not demonstrate decreased pSAMD5^+^ cell numbers when compared to the MRL/MpJ-Sham mice. The MRL/MpJ-OV mice showed significantly greater numbers of pSMAD5^+^ cells than the WT-OV mice (*p* = 0.01) (Figure 8b,f).

Immunohistochemistry staining demonstrated PCNA^+^ cells located both on the bone surface and within the bone marrow. The number of PCNA^+^ cells on the bone surface was decreased in the WT-OV group when compared to the WT-Sham group (*p* = 0.01). No statistical difference for PCNA^+^ cells was observed between the MRL/MpJ-OV mice and the MRL/MpJ-Sham mice. The PCNA^+^ cells of the MRL/MpJ-OV mice were also significantly greater than that of the WT-OV mice (*p* < 0.05) (Figure 8c,g).

TRAP staining was further utilized to identify osteoclasts in the proximal tibia trabecular bone. TRAP^+^ cells were stained violet-red on the bone surface with single or multiple nuclei. The WT-OV and MRL/MpJ-OV mice did not show any significant differences in the number of TRAP^+^ cells compared to their sham counterparts. However, the MRL/MpJ-Sham and MRL/MpJ-OV mice showed significantly lower TRAP^+^ cells on the bone surface compared to their WT counterparts (*p* < 0.01 and 0.05, respectively) (Figure 8d,h). TRAP staining was also performed for the vertebral tissues where very few TRAP^+^ cells were identified on the spine trabecular bone with no differences between any groups.

### 2.8. Serum Protein ELISA and Multiplex Measurements after Ovariectomy

**Figure 9:**
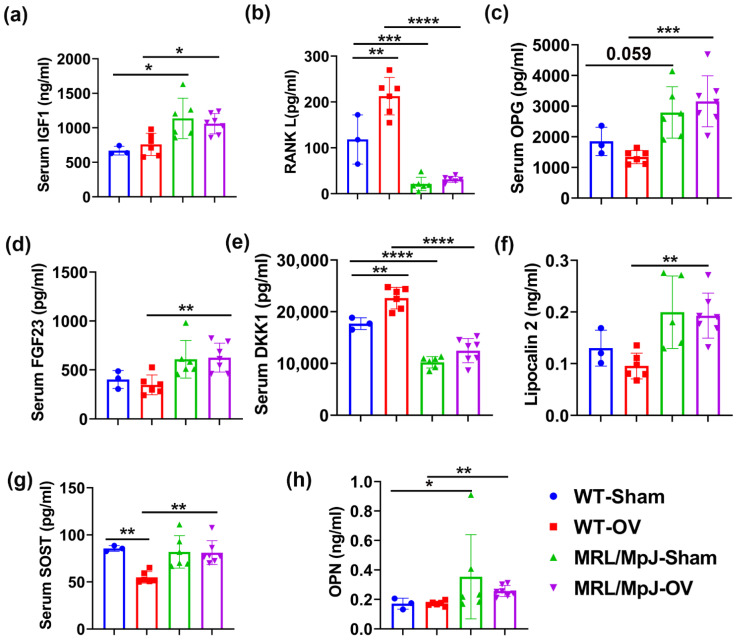
Serum level of bone metabolism factors at 6 M after surgery. (**a**) Serum IGF1 level. The IGF1 level in MRL/MpJ-Sham and MRL/MpJ-OV groups was significantly higher than that of WT-Sham and WT-OV mice, respectively. (**b**) RANKL level. WT-OV mice showed significantly higher serum levels of RANKL than WT Sham mice. MRL/MpJ-Sham and MRL/MpJ-OV mice showed significantly lower serum RANKL levels than the WT-Sham and WT-OV mice, respectively. (**c**) Serum OPG level. The serum OPG levels in MRL/MpJ-Sham and MRL/MpJ-OV mice were significantly higher than those in the WT-Sham (*p* = 0.059) and WT-OV groups, respectively. (**d**) Serum FGF23 level. MRL/MpJ-OV mice showed significantly higher levels of FGF23 than WT-OV mice. (**e**) Serum DKK1 level. The DKK1 level was significantly increased in WT-OV mice compared to WT-Sham mice. The DKK1 level was significantly lower in MRL/MpJ-Sham mice and MRL/MpJ-OV mice compared to WT-Sham and WT-OV mice, respectively. (**f**) Serum lipocalin 2 level. The serum lipocalin 2 levels were significantly higher in MRL/MpJ-OV mice than in WT-OV mice. (**g**) Serum SOST level. WT-OV showed decreases in the SOST level compared to WT-Sham and MRL/MpJ-OV mice, respectively. (**h**) Serum OPN level. The serum OPN level in MRL/MpJ-Sham mice and MRL/MpJ-OV mice was significantly higher than that of WT-Sham and WT-OV mice. * *p* < 0.05, ** *p* < 0.01, *** *p* < 0.001, **** *p* < 0.0001.

ELISA demonstrated that the IGF1 level in the MRL/MpJ-Sham group was significantly higher than that of the WT-Sham (*p* < 0.05). The IGF1 level was also significantly higher in the MRL/MpJ-OV group than in the WT-OV group (*p* < 0.05) (Figure 9a). Regarding RANKL, the WT-OV mice showed significantly higher serum RANKL levels compared to the WT-Sham mice (*p* < 0.01), while the MRL/MpJ-OV mice did not show significant differences in RANKL levels after ovariectomy. The MRL/MpJ-Sham mice showed significantly lower serum RANKL levels than the WT-Sham mice (*p* < 0.001). The MRL/MpJ-OV mice also showed significantly lower levels of RANKL as compared to WT-OV mice (*p* < 0.0001) (Figure 9b). The multiplex assay results demonstrated that serum OPG in the MRL/MpJ-Sham mice was higher than in the WT-Sham (*p* = 0.059). The serum OPG level in the MRL/MpJ-OV mice was also significantly higher than in the WT-OV mice (*p* < 0.001) (Figure 9c). MRL/MpJ-OV also showed significantly higher levels of FGF23 than the WT-OV mice (*p* < 0.01), while no differences were found between other groups (Figure 9d). The DKK1 level was found to be significantly increased in the WT-OV mice compared to the WT-Sham mice (*p* < 0.01). However, the serum DKK1 level was significantly lower in the MRL/MpJ-Sham mice compared to the WT-Sham (*p* < 0.0001). The DKK1 level of the MRL/MpJ-OV mice was also significantly lower compared to the WT-OV mice (*p* < 0.0001) (Figure 9e). The serum lipocalin 2 level in the MRL/MpJ-OV mice was significantly higher than in the WT-OV mice (*p* < 0.01). Furthermore, the WT-OV mice showed significantly lower levels of SOST compared to the WT-Sham (*p* < 0.01); however, the serum SOST level of MRL/MpJ-OV mice was significantly higher than that of WT-OV mice (*p* < 0.01) (Figure 9g). The serum OPN level in the MRL/MpJ-Sham mice was significantly higher than the level in the WT-Sham mice (*p* = 0.05). The OPN level in the MRL/MpJ-OV mice was significantly higher than that of the WT-OV mice (*p* < 0.01) (Figure 9h). No statistical differences were found between groups for other factors measured.

## 3. Discussion

The main findings of the study were that MRL/MpJ mice maintained a higher bone mass up to 14-months-old as revealed by micro-CT when compared to WT mice. The micro-CT results are further reinforced by histology findings such as COL1 content, as revealed by Herovici’s staining. The higher bone mass observed in MRL/MpJ mice, independent of sex, correlates with higher OSX^+^ osteoprogenitor cells; higher levels of pSMAD5 activation, an important signaling pathway for bone formation and osteogenic differentiation; and, lastly, a high number of PCNA^+^ cells. Furthermore, serum IGF-1, OPG, FGF23, OPN, SOST, and lipocalin 2 are significantly higher in male and female MRL/MpJ mice, while RANKL and DKK1 are significantly lower, compared to their WT counterparts. The MRL/MpJ mice are also resistant to long-term ovariectomy-induced bone loss, as demonstrated by micro-CT and histology. In fact, MRL/MpJ-OV mice maintained higher OSX^+^, pSMAD5^+^, and PCNA^+^ cell numbers than their WT counterparts despite having a reduction in OSX^+^ cell numbers compared to MRL/MpJ-Sham mice. Additionally, the MRL/MpJ-OV mice maintained higher levels of serum IGF1, OPG, and lipocalin 2, and lower levels of RANKL and DKK1, when compared to WT mice (with or without ovariectomy). WT mice showed increases in bone catabolism factors, RANKL and DKK1, after ovariectomy compared to WT-Sham. In contrast, MRL/MpJ mice did not show increased serum levels of RANKL and DKK1 after ovariectomy.

Age-related bone loss causes osteoporosis and results in an increased risk of fractures, especially vertebral fractures [3,34]. This study sought to investigate if MRL/MpJ mice also maintained higher bone microarchitecture during aging, as reported previously at younger ages [24], and uncover potential anabolic factors that contribute to the resistance to age-related bone loss in MRL/MpJ mice. Indeed, our micro-CT and histology results demonstrated significantly higher bone microarchitecture in MRL/MpJ mice than in WT mice for both males and females. Interestingly, we have also observed that female MRL/MpJ mice have similar or even better bone microarchitecture parameters than their male counterparts, in contrast to worse bone parameters observed in female WT mice compared to male WT mice. These results are even more obvious for the vertebral bones. Mechanistically, we found significantly higher numbers of OSX^+^, pSMAD5^+^, and PCNA^+^ cells and relatively fewer SOST^+^ cells in male and female MRL/MpJ mice. Therefore, a combination of the higher number of OSX^+^ cells, higher-level activation of pSMAD5 signaling pathways, and cell proliferation in MRL/MpJ mice likely contributes to the maintenance of enhanced bone microarchitecture during aging. Previously, we have shown that MRL/MpJ mice have higher OCN^+^ osteoblasts and PCNA^+^ cells on the bone surface at younger ages [24]. These factors likely also play important roles in maintaining enhanced bone microarchitecture during aging, as demonstrated in this study.

To investigate which factors drive the maintenance of the osteogenic progenitor pool in MRL/MpJ mice, we further performed ELISA and multiplex assays on the sera of these mice. We observed significantly higher serum levels of IGF1, OPG, FGF23, OPN, SOST, and lipocalin 2 and lower levels of RANKL and DKK1 in 14-month-old MRL/MpJ mice compared to WT mice. Since it was previously reported that in younger MRL/MpJ mice, serum IGF1 levels were found to be significantly higher and RANKL levels significantly lower than in WT mice [24], we believe that increased IGF1 and decreased RANKL also play an important role in maintaining better bone microarchitecture during the aging process in MRL/MpJ mice.

Circulating IGF1 is essential for bone growth and an important bone anabolic factor [35]. IGF1 released from the bone matrix during bone remodeling stimulates bone formation via the activation of the mTOR signaling pathway [36]. In support of this contention, the IGF-1 levels in bone matrix and bone marrow were also found to be decreased in aged rats and humans, while the infusion of IGF1+IGFBP3 increased bone matrix IGF1, which consequently stimulated bone formation in aged rats [36]. The role of serum IGF1 levels in age-related osteoporosis remains controversial. Several studies showed lower serum IGF1 levels in osteoporotic patients and that the lower level of IGF1 correlated with an increased risk of sustaining osteoporotic fractures [37,38,39,40,41,42,43]. Another study demonstrated that higher IGF1 levels were correlated with lower bone formation rates in idiopathic osteoporotic patients [44]. In addition, it has been also reported that both increased and decreased IGF1 levels can be detrimental to bone formation [45]. We believed that higher IGF1 levels in MRL/MpJ mice contributed to the maintenance of bone mass during the aging process, potentially by increasing OSX^+^ osteogenic progenitors via activation of the IGF1-mTOR-pAKT pathway.

Since our study also revealed that serum RANKL was decreased, and OPG, a decoy receptor of RANKL, was increased in MRL/MpJ mice, we assume that OPG is another potential contributor to the maintenance of bone microarchitecture during the aging process in MRL/MpJ mice. Given that lower RANKL and higher OPG levels resulted in an even lower RANKL/OPG ratio, we believe that the RANKL/OPG axis potentially contributed to the lower osteoclastogenesis observed in the MRL/MpJ mice. RANKL, secreted by osteoblasts or osteocytes, is an important growth factor for promoting osteoclastogenesis and bone remodeling and a key player in the balance of bone formation and remodeling [46]. In fact, the FDA-approved RANKL antibody, Denosumab, has been used in clinical practice as an anti-resorptive drug for treating osteoporosis [47,48,49]. Therefore, the finding of the current study that MRL/MpJ mice, independent of sex, showed lower serum RANKL levels and higher OPG levels, compared to WT mice, supports the notion that these factors are major contributors to the maintenance of bone microarchitecture during aging in MRL/MpJ mice.

DKK1 and SOST are both Wnt signaling inhibitors [33]. The serum levels of DKK1 in postmenopausal women were significantly higher than in controls, which correlates negatively with lumbar spine and femur T-scores as well as the duration of menopause [50]. Higher serum DKK1 is also negatively correlated with β-catenin and OPG [51]. Multivariant analysis has shown that serum levels of DKK1 negatively correlate with beta-catenin and BMD and positively correlate with the bone turnover marker CTX [51]. Higher levels of serum DKK1 correlate more with bone erosion in patients with systemic lupus erythematosus (SLE), who are anti-CCP-positive, than in those with either non-erosive arthritis or without arthritis [52].

DKK1 levels are significantly increased in patients with SOST deficiency [53]. DKK1 levels are significantly higher in patients with both sclerosteosis and van Buchem disease (VBD) when compared to levels in carriers of the two diseases (sclerosteosis) and healthy controls [53]. Serum DKK1 levels are positively associated with levels of procollagen type 1 amino-terminal propeptide and carboxy-terminal cross-linked telopeptide in both disorders [53]. This is considered an adaptive response to the high bone formation in SOST-deficient patients [53]. Another study showed that in patients older than 75 years who sustained a hip fracture, serum SOST and DKK1 levels were significantly higher when compared to healthy controls [54]. Furthermore, a study performed in Korea showed lower plasma SOST levels observed in osteoporotic patients presenting with a fracture than in controls without fractures. The odds ratio of osteoporotic fracture is 2.97 in patients with a lower SOST percentile than a higher SOST percentile; however, DKK1 plasma levels did not correlate with osteoporotic fracture risk [55]. DKK1 expression can be triggered by TNFα in TIMP-null mice through the TIMP/MMPs–TNFα–RANKL nexus [56]. A previous study showed that DKK1 and SOST levels in the cortical bone matrix correlated positively with bone mass and strength in postmenopausal women with osteoporosis [57]. DKK1 induced by 1,25-vitamin D3 is required for osteoblast-induced mineralization through the activation of C/EBPβ [58]. DKK1 deficiency in osteoprogenitor cells or osteocytes protects against glucocorticoid-induced bone loss but not arthritis-induced bone loss [59]. Knocking out DKK1 in T-cells protects against ovariectomy-induced bone loss via increasing bone formation and decreasing osteoclasts [60]. Indeed, the systemic neutralization of DKK1 enhanced the human adipose-derived stem-cell-mediated repair of femoral segmental bone defects by increasing cell engraftment, survival, and vascular ingrowth in the newly formed bone [60]. However, one study reported that bone DKK1 and SOST positively correlated with bone mineral density, microarchitecture, and strength in postmenopausal osteoporosis [61]. Our study showed that serum DKK1 decreased while SOST increased in MRL/MpJ mice during aging when compared to WT mice. Since both factors are inhibitors of Wnt-β-catenin signaling, the net effect is that these two factors may play a major role in the maintenance of high bone mass in MRL/MpJ mice during aging.

Our study also found that MRL/MpJ mice showed lower BV/TV and Tb.N loss percentages than the WT mice after ovariectomy surgery, as demonstrated by micro-CT and histology in both vertebrae and long bones. We further demonstrated that after ovariectomy, WT mice showed a decrease in OSX^+^ cells, pSMAD5^+^ cells, and PCNA^+^ cells compared to WT-Sham mice. However, MRL/MpJ mice only demonstrated a decrease in OSX^+^ cells after ovariectomy compared to MRL/MpJ-Sham mice. These results indicate that ovariectomy-induced bone loss due to estrogen deficiency might be compensated for by the intrinsic mechanism that maintains the higher bone mass of MRL/MpJ mice. Indeed, our study revealed that RANKL and DKK1 were further increased, SOST was further decreased in WT mice after ovariectomy compared to WT sham mice. Ho RANKL and SOST levels did not change in MRL/MpJ mice after ovariectomy. This finding was consistent with a previous study showing that RANKL significantly increased after ovariectomy in rats [62]. No study reports DKK1 changes after ovariectomy, and, therefore, maintaining lower levels of RANKL and DKK1 in MRL/MpJ mice may contribute to their resistance to bone loss after ovariectomy surgery. Furthermore, MRL/MpJ mice maintained high levels of IGF1, OPG, and lower levels of RANKL and DKK1 after ovariectomy compared to WT mice after ovariectomy surgery. These factors likely also contribute to less severe bone loss in the MRL/MpJ mice after ovariectomy. Of note, MRL/MpJ mice after ovariectomy also showed Tb.Th loss despite significantly lower Tb.N loss, suggesting that estrogen levels do not play a role in the maintenance of higher bone mass after ovariectomy in MRL/MpJ mice.

## 4. Materials and Methods

### 4.1. Animal Use Ethics

All mice usage and procedures were approved by the University of Texas Health Science Center Institutional Animal Care and Use Committee (AWC-16-0132). As the life span of MRL/MpJ mice is between 73 and 93 weeks, 14-month-old (60-week-old) mice were chosen for this study. Male and female MRL/MpJ (Cat#:000486) and C57BL/6J (WT, Cat#:000664) mice were purchased from Jackson Laboratories (Bar Harbor, ME, USA) and were bred in the animal facility of the University of Texas Health Science Center at Houston to produce appropriately aged mice for this study.

### 4.2. Characterization of the Bone Microarchitecture of MRL/MpJ and WT Mice at 14 M

Based on the power analysis of micro-CT, N = 8 is sufficient to detect differences without considering gender differences. We have included more MRL/MpJ mice to increase the confidence of conclusions. WT-F and WT-M mice were fewer because the litter size is smaller. MRL/MpJ-F (N = 10 mice) and MRL/MpJ-M mice (N = 8), as well as WT-F (N = 3) and WT-M (N = 5) mice, were aged and sacrificed at 14 months of age. Spines, right tibial, and femoral tissues were harvested and fixed in neutral buffered formalin (NBF, Sigma-Aldrich Inc., St. Louis, MO, USA) for micro-CT scanning and histological analysis. Serum was also collected for serum protein analysis.

### 4.3. Micro-CT Scanning and Analysis of 14 M Mice Bone Specimens

After fixation, mouse bone tissues were wrapped in parafilm and scanned using Viva CT 40 (SCANCO Medical AG, Fabrikweg, Switzerland) using 70 kVP and 114 μA and 15 μm voxel size (resolution). The cancellous bone of spine L5 was analyzed by manually contouring and using morph functions to include the entire spine L5 bone, utilizing the manufacturer’s built-in 3D evaluation software. The analysis parameters were Gauss 1, sigma = 0.8, and a threshold of 163. The bone parameters, including BV/TV, Tb.N, Tb.Th, and Tb.Sp, were automatically generated by 3D analysis software V6.6. For the proximal tibia trabecular bone, we chose 50 slices below the growth plate (metaphysis) for analysis by using manual contouring and morph functions to define the view of interest. The analysis parameters were the same as those for the spine L5 trabecular bone, as stated above. For the analysis of femoral cortical bone, 50 slices of midshaft femur were chosen and analyzed using the parameters of Gauss 1, sigma = 0.8, and threshold 200. The Ct.Th (mm) and cortical BV density were automatically generated by the software.

### 4.4. Ovariectomy Surgeries

MRL/MpJ and WT female mice at the age of 4 M were divided into WT-Sham (N = 4), WT-OV (N = 6), MRL/MpJ-Sham (N = 6), and MRL/MpJ-OV (N = 8) groups. Mice were anesthetized using 2–3% isoflurane and subjected to sham surgery or ovariectomy by removing both ovaries, according to a previously published protocol [63]. After surgery, mice recovered on a warm pad and were housed ad libitum for 6 M.

### 4.5. Micro-CT Scanning and Analysis of Mice Undergoing Ovariectomy and Sham Surgery

Mice were scanned for the spine L4–6 and proximal tibia at day 1 and 1, 2, 4, and 6 M after ovariectomy or sham surgery using Viva CT40 (SCANCO Medical AG, Fabrikweg, Switzerland). Mice were scanned using 70 kVP and 114 μA and 30 μm voxel size (medium resolution) under 2–3% anesthesia. After the last micro-CT in vivo scanning at 6 M, blood was collected using a retroorbital approach prior to sacrificing while mice were still under anesthesia. Mice were then sacrificed by cervical dislocation. Sera were isolated by centrifugation. The right-side tibia and femur, in addition to the lumbar spine, were harvested and fixed in NBF for 3 days for histology.

### 4.6. Histology

All bone specimens were decalcified using 10% ethylenediaminetetraacetic acid disodium (EDTA) (Sigma-Aldrich Inc., St. Louis, MO, USA) plus 1% sodium hydroxide (NaOH) (pH = 7.0) (Fisher Scientific, Waltham, MA, USA) for 1 month. Tissues were then processed using gradient ethanol and xylene and were then paraffin-embedded. Sections of 5 μm were cut with a microtome for histology staining and immunohistochemistry. H&E staining was performed using ANATECH reagents (ANATECH LTD, Battle Creek, MI, USA) following the manufacturer’s protocol. Herovici’s staining was performed as previously described [64,65,66] to differentiate COL 1 and COL 3. Approximately 95% bone matrix is COL1. All staining reagents were purchased from Sigma (Sigma-Aldrich Inc., St. Louis, MO, USA). TRAP staining was performed using an Acid Phosphatase, Leukocyte (TRAP) Kit (387A-1KT, Sigma-Aldrich Inc., St. Louis, MO, USA) following the manufacturer’s protocol. TRAP^+^ cells on the bone surface were quantified using Image J (NIH) by counting positive cell numbers and measuring the bone perimeters of each image. Quantification was expressed as TRAP^+^ cells/mm bone surface.

### 4.7. Immunohistochemistry

Immunohistochemical staining for OSX, pSMAD5, PCNA, and SOST was performed as previously described [24,25,65,67]. Briefly, paraffin sections were deparaffinized and rehydrated in water. Different antigen retrieval methods were used before blocking and primary antibody incubation. For OSX (ab 22552, 1:1000 dilution, Abcam, Waltham, MA, USA), no antigen retrieval was needed. For pSMAD5 (ab92698, 1:400 dilution, Abcam, Waltham, MA, USA) and PCNA (ab18197, 1:4000 dilution, Abcam, Waltham, MA, USA), heat-mediated antigen retrieval was performed using 10 mM citrated buffer (pH = 6) at 92 °C for 20 min and cooled for 10 min. For SOST (AF1589, 1:50 dilutions R&D system Inc., Minneapolis, MN, USA), antigen retrieval was performed using 10 ng/mL proteinase K in Tris-EDTA buffer (pH = 8.0) at 37 °C for 20 min. All sections were blocked with 5% donkey serum for 1 h at room temperature and then incubated with primary antibodies at 4 °C overnight. The slides were then washed with phosphate-buffered saline (PBS) and treated with 0.5% hydrogen peroxide in PBS for 30 min at room temperature. After another wash, slides were then incubated with a biotinylated secondary antibody for 2 h at room temperature. For OSX, pSMAD5, and PCNA, goat anti-rabbit-biotin (BA-1000, Vector Laboratories Inc., Newark, CA, USA) was used at a 1:300 dilution. For SOST, horse anti-goat-biotin (BA-9500-1.5, Vector Laboratories Inc., Newark, CA, USA) was used at a 1:300 dilution. After a PBS wash, the slides were then incubated with VECTASTAIN^®^ Elite ABC-HRP Kit (Peroxidase, Universal) (Vector Laboratories Inc., Newark, CA, USA ) for another 2 h (premixed A and B 30 min before applying to slides). After PBS wash, a DAB kit (SK-4100) or NovaRed kit (SK-4800, Vector Laboratories Inc., Newark, CA, USA) was used for color reaction to reveal positive cells. After rinsing with water, hematoxylin QS (H-3404-100, Vector Laboratories Inc., Newark, CA, USA) was used to counterstain the cell nuclei. Slides were then dehydrated in gradient alcohol, cleared with xylene, dried, and a coverslip was then applied with Cytoseal (Fisher Scientific, Waltham, MA, USA) mounting medium. Images were captured with a Nikon CIL microscope (Nikon Instruments Inc., Melville, NY, USA). For quantification, the entire spine L6, proximal tibia, or midshaft femoral cortical bone were imaged at 200× magnification. Positive cell numbers and bone surface or area were counted and measured using Image J software. The positive cells were expressed as cell number/mm bone surface or cell number/mm^2^ bone area.

### 4.8. Serum Bone-Related Protein Analysis

Blood was collected using a retro-orbital collection method. Sera were isolated by centrifugation and stored at −80 °C until analysis with ELISA kits or multiplex panels. IGF1 was measured with Mouse/Rat IGF-I/IGF-1 Quantikine ELISA Kit (MG100, R&D system Inc., Minneapolis, MN, USA). RANKL was measured with Mouse TRANCE/RANK L/TNFSF11 Quantikine ELISA Kit (MTR00, R&D system Inc., Minneapolis, MN, USA). Fibroblast growth factor 21 (FGF21) was measured with Mouse/Rat FGF-21 Quantikine ELISA Kit (MF2100, R&D system Inc., Minneapolis, MN, USA). Periostin was measured with Mouse Periostin/OSF-2 Quantikine ELISA Kit (MOSF20, R&D system Inc., Minneapolis, MN, USA). All ELISAs were performed following the manufacturer’s manual and absorbance was measured with the Tecan Infinite 200 microplate reader (Tecan Group Ltd., Männedorf, Switzerland). The mouse bone panel (Cat.#:MBNMAG-41K, MilliporeSigma, Burlington, MA, USA) and mouse kidney injury panel 2 (MKI2MAG-94K, MilliporeSigma, Burlington, MA, USA) multiplex were used to measure other factors in the serum following the manufacturer’s protocol. The mouse bone panel included adrenocorticotropic hormone (ACTH), DKK1, FGF23, interleukin 6 (IL6), insulin, leptin, OPG, SOST, and tumor necrosis factor α (TNFα). The mouse kidney injury panel 2 included clusterin, cystatin C, epidermal growth factor (EGF), NGAL/lipocalin 2, and OPN. Magnetic bead fluorescence intensity was detected with Luminex^®^ 100/200™ System (Luminex Coportate, Austin, TX, USA).

### 4.9. Statistical Analysis

Statistical analysis was performed using one-way analysis of variance using Graphpad Prism 9.5.0 (Graphpad Software, Boston, MA, USA) followed by Tukey’s post hoc multiple comparisons tests for micro-CT and serum marker quantification. We used T-tests for histology parameter comparison because we were only interested in the comparison of males and females of the same strains, WT-F and MRL/MpJ females or WT-M and MRL/MpJ males. If the SD was high, we used a two-group Wilcoxon Rank Sum test. *p* < 0.05 was considered statistically significant.

## 5. Conclusions

In summary, our results demonstrated that MRL/MpJ mice maintained higher bone microarchitecture during aging and following ovariectomy. Histologically, the greater number of OSX^+^, pSMAD5^+^, and PCNA^+^ cells and the lower number of TRAP^+^ cells contributed to the greater bone mass in the MRL/MpJ mice. Systemically, high serum IGF1 and OPG and lower RANKL and DKK1 may play an important role in MRL/MpJ mice’s ability to resist age-related bone loss and less severe bone loss due to long-term ovariectomy. These findings may be used to develop therapeutic approaches to maintain bone mass and improve bone regeneration and repair after injury, disease, and aging.

## Data Availability

The data presented in this study are available upon request to the corresponding author.

## Supplementary Materials

The following supporting information can be downloaded at: https://www.mdpi.com/article/10.3390/ijms24032396/s1.
