# Supplementary material for: MRL/MpJ Mice Resist to Age-Related and Long-Term Ovariectomy-Induced Bone Loss: Implications for Bone Regeneration and Repair
Source: Int J Mol Sci. 2023 Jan 25;24(3):2396. doi: 10.3390/ijms24032396 (PMC9916619; doi:10.3390/ijms24032396)
Supplement: Supplementary file 1 [file ijms-24-02396-s001.zip › ijms-2102851-supplementary.pdf]

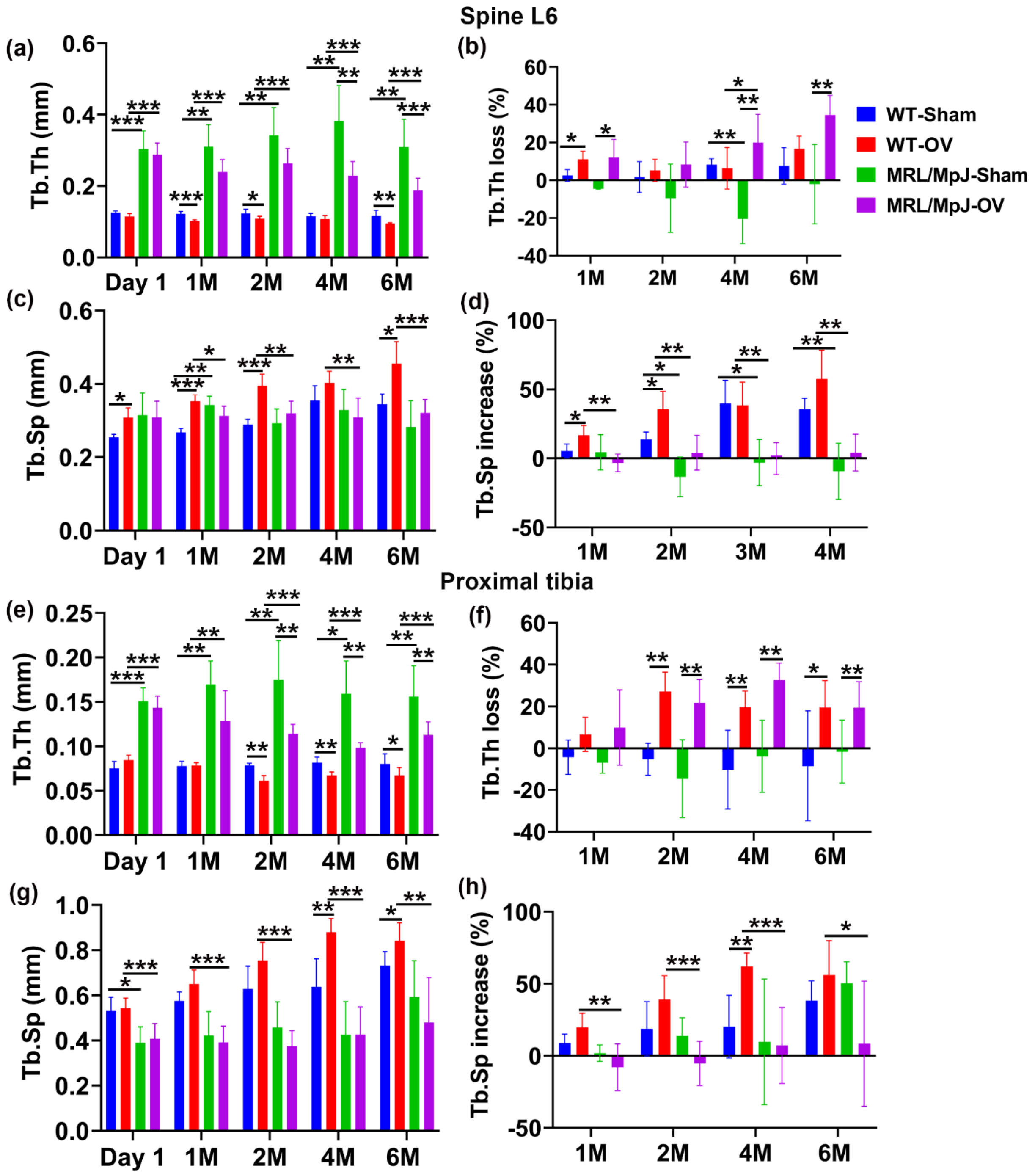

Supplemental figure S1: Spine L6 and proximal tibia Micro-CT Tb.Th and Th.Sp changes after ovariectomy. (a) Tb.Th of spine L6 after ovariectomy. The Tb.Th of MRL/MpJ-Sham and MRL/MpJ-OV mice maintained consistently higher values than the WT-Sham and WT-OV groups respectively after ovariectomy. At 1, 2 and 6 M after ovariectomy, the Tb.Th of WT-OV mice showed significant decreases compared to WT-Sham mice ($P<0.001$, 0.05 and 0.01, respectively). At 4 and 6 months, MRL/MpJ-OV mice showed significant decreases in Tb.Th compared to the MRL/MpJ-Sham mice ($P<0.01$ and 0.001 respectively). (b). Tb.Th loss

percentage of spine L6. At 1 month, both WT-OV and MRL/MpJ-OV showed significant greater Tb.Th loss percentages as compared to those of the WT-Sham and MRL/MpJ-Sham groups, respectively (both P<0.05). At 4M, MRL/MpJ-OV mice showed significantly greater Tb.Th loss percentage than the WT-OV mice as well as the MRL/MpJ-Sham mice. However, MRL/MpJ-Sham mice did not show Tb.Th loss and showed significantly less Tb.Th loss percentage than WT-Sham. At 6M, MRL/MpJ-OV mice showed significantly greater Tb.Th loss percentage than MRL/MpJ-Sham mice. No statistical differences were found among other groups. (c). Tb.Sp increase of spine L6. At 1M, WT-OV mice demonstrated significantly increased Tb.Sp compared to WT-Sham mice. MRL/MpJ-OV did not demonstrate increases in Tb.Sp and showed significantly lower Tb.Sp compared to WT-OV. At 2M, WT-OV showed significantly larger Tb.Sp than that of WT-Sham. MRL/MpJ-OV mice showed significantly less Th.Sp compared to WT-OV mice. At 4M, MRL/MpJ-OV also showed significantly lower Tb.Sp than WT-OV mice. At 6M, WT-OV showed significantly higher Tb.Sp than that of WT-Sham. MRL/MpJ-OV showed significantly lower Tb.Sp than that of WT-OV mice. (d). Tb.Sp increase percentages. The Tb.Sp increase percentages in MRL/MpJ-OV mice did not show significantly elevated at any time points after OV. The Tb.Sp increase percentage of WT-OV showed significant increases at 1 and 2 months after surgery compared to WT-Sham mice (P<0.05 for both). MRL/MpJ-OV mice showed significantly less Tb.Sp increases percentage than those of WT-OV mice at 1, 2, 4 and 6M. MRL/MpJ-Sham mice did not show Tb.Sp increases and demonstrated significantly less Tb.Sp increase percentages at 2, 4 and 6M. (e) Tb.Th of proximal tibia at different time points after ovariectomy. Both MRL/MpJ-Sham and MRL/MpJ-OV showed significantly greater Tb.Th than those of WT-Sham and WT-OV at all time points. At 2M, 4M and 6M, WT-OV showed significantly decrease in Tb.Th than those of WT-Sham (All are statistical difference). At 2, 4 and 6M, MRL/MPJ-OV mice also showed significantly lower Tb.Th than those of MRL/MpJ-Sham mice (all P<0.01). (f) Tb.Th loss percentage of proximal tibia after ovariectomy compared to day 1. Both WT-Sham and MRL/MpJ-Sham did not show significant Tb.Th losses. WT-OV showed significantly more Tb.Th loss percentage than WT-Sham mice at 2M,4M and 6 M (All had statistical difference). MRL/MpJ-OV mice showed significantly more Tb.Th loss than those of WT-Sham and MRL/MpJ-Sham at 2M,4M and 6M after ovariectomy (P<0.01 for all comparisons). (g) Tb.Sp of proximal tibia after ovariectomy. MRL/MpJ-Sham and MRL/MpJ-OV showed significantly lower Tb.Sp at day 1 that those of WT-Sham and WT-OV (P<0.05 and P<0.001, respectively). WT-OV mice showed significantly greater Tb.Sp at 4M compared to that of WT-Sham mice (P<0.01). MRL/MpJ-OV mice showed significantly lower Tb.Sp than that of WT-OV mice at all time points after ovariectomy. (h). Tb.Sp increase percentage of proximal tibia after ovariectomy. Tb.Sp increase percentage was significantly higher in WT-OV mice than those WT-Sham mice (P<0.01) at 4 months after surgery. MRL/MpJ-OV mice did not show significant Tb.Sp increase percentages as compared to the MRL/MpJ-Sham mice. However, MRL/MpJ-OV mice showed significantly less Tb.Sp increase percentage than that of WT-OV at 1, 2, 4 and 6 months after surgery. *P<0.05, **P<0.01, ***P<0.001.
